# Genetics, leadership position, and well-being: An investigation with a large-scale GWAS

**DOI:** 10.1073/pnas.2114271119

**Published:** 2022-03-14

**Authors:** Zhaoli Song, Wen-Dong Li, Xuye Jin, Junbiao Ying, Xin Zhang, Ying Song, Hengtong Li, Qiao Fan

**Affiliations:** ^a^Department of Management and Organization, National University of Singapore, 119245, Singapore;; ^b^Department of Management, The Chinese University of Hong Kong, Shatin, N.T., Hong Kong, China;; ^c^Department of Statistics and Data Science, National University of Singapore, 117546, Singapore;; ^d^Centre for Quantitative Medicine, Duke-NUS Medical School, 169857, Singapore

**Keywords:** GWAS, leadership, well-being, genetics, management

## Abstract

Twin studies document leadership role occupancy (e.g., whether one holds formal supervisory or management positions) as a heritable trait. However, previous studies have been underpowered in identifying specific genes associated with this trait, which has limited our understanding of the genetic correlations between leadership and one’s well-being. We conducted a genome-wide association study (GWAS) on individuals’ leadership phenotypes that were derived from supervisory/managerial positions and demands among 248,640 individuals of European ancestry from the UK Biobank data. Among the nine genome-wide significant loci, the identified top regions are pinpointed to previously reported GWAS loci for bipolar disorder (*miR-2113/POUSF2* and *LINC01239*) and schizophrenia loci (*ZSWIM6*). We found positive genetic correlations between leadership position and several positive well-being and health indicators, including high levels of subjective well-being, and low levels of anxiety and depression (|*r_g_*| > 0.2). Intriguingly, we observed positive genetic correlations between leadership position and some negative well-being indicators, including high levels of bipolar disorder and alcohol intake frequency. We also observed positive genetic correlations between leadership position and shortened longevity, cardiovascular diseases, and body mass index after partialing out the genetic variance attributed to either educational attainment or income. The positive genetic correlation between leadership and bipolar disorder seems potentially more pronounced for those holding senior leadership positions (*r_g_*: 0.10 to 0.24), partially due to shared genetic variants with educational attainment. Our findings provide insights into the polygenic nature of leadership and shared genetic underpinnings between the leadership position and one’s health and well-being. We caution against simplistic interpretations of our findings as advocating genetic determinism.

## Significance

Our study presents the largest whole-genome investigation of leadership phenotypes to date. We identified genome-wide significant loci for leadership phenotypes, which are overlapped with top hits for bipolar disorder, schizophrenia, and intelligence. Our study demonstrated the polygenetic nature of leadership, the positive genetic correlations between leadership traits and a broad range of well-being indicators, and the unique association of leadership with well-being after accounting for genetic influences related to other socioeconomic status measures. Our findings offer insights into the biological underpinnings of leadership.

Leadership has been demonstrated to have profound influences on the performance and well-being of individuals, groups, organizations, and the world. One school of thought in leadership research—inspired by the Great Man theory (1)—focuses on the role of individual characteristics that distinguish leaders from nonleaders. Such individual characteristics include intelligence, personality, and physical height (2, 3), which are heritable (4, 5). Twin studies reported a heritability estimate of ∼30% for leadership role occupancy (6, 7), that is, whether one holds leadership positions or not. There have also been some recent efforts to identify specific genes that may be related to holding leadership positions (8, 9). Yet we currently know little about genetic variants that may modulate leadership at the whole-genome scale. This has, in turn, limited our understanding of how leadership and other important outcomes, such as health and well-being variables, are genetically related.

The management literature has a long history of examining the phenotypic relationship between leadership and job incumbents’ well-being (10–12). An important reason is that leaders’ well-being, as an important issue in and of itself, affects leaders’ behaviors, which may in turn influence the performance and well-being of their subordinates, teams, and organizations. As such, gaining a deeper understanding of to what extent leadership role occupancy is shaped by the genetic lottery will advance our knowledge of the nature and the etiology of the phenotypic relationship between leadership and well-being. Such an enriched understanding, in turn, has implications for leaders and prospective leaders in managing their health and well-being for their long-term career development. At the phenotypic level, early research—with a handful of evidence—alludes to some detrimental influences of leadership role occupancy on one’s health because of abundant job responsibilities embedded in the leadership position (13, 14). More recent studies have shown that being a leader—as an indicator of high socioeconomic status (SES)—may be beneficial to one’s health due to the resources inherent in leadership positions (12, 15). Adding another layer of complexity, some recent reports have also shown a positive correlation of the leadership position with bipolar disorder (16) or alcohol consumption (17). Examining genetic correlations between leadership and well-being variables may shed light on these mixed phenotypic findings in terms of whether being a leader is beneficial or detrimental to one’s well-being at the genetic level. Furthermore, given early reports of substantial genetic correlations between well-being and other SES indicators, including educational attainment and income (18, 19), it is worthwhile to investigate whether leadership position has a unique contribution to well-being beyond these SES measures.

The main goal of this research is twofold. First, we conducted a genome-wide association study (GWAS) of two leadership variables—leadership position and managing demands—with the UK Biobank (UKB) data and replicated top variants in three independent samples. Due to the multifaceted nature of the leadership construct, we adopted the approach in leadership research from the management and behavioral genetics and focused on the two leadership phenotypes with a focus on holding a leadership position and performing critical managing functions (6, 20). Leadership research suggests that holding formal leadership positions grants one with great opportunities to influence others, which is at the core of the definition of leadership (21); it also represents the “first step” in one’s leadership processes to advance his or her career (4). Accordingly, it was measured as whether one held supervisory or managerial occupations, which was derived from the UK Standard Occupation Classification (SOC). Managing demands refer to the amount of critical leadership functions needed to manage other people, resources, and work tasks regardless of one’s holding a formal leadership position or not. Information on managing demands was derived from items measuring managerial responsibilities in the US Occupational Information Network (O*NET). Together, the two leadership variables are able to capture a broader spectrum of leadership situations in which one may hold formal or informal leadership positions (or the lack thereof). Second, we examined genetic correlations of the leadership phenotypes with personality traits and a number of well-being indicators before and after partialing out the genetic variance in common with educational attainment and income. This allows us to showcase the unique role of leadership in such relationships as a distinctive SES variable related to one’s occupational achievement.

## Results

### Phenotypical Measures of Leadership Traits.

Management research has defined leadership as a process of influencing individuals in a group or an organization to move toward a common goal (21). Given the importance of holding a leadership position in the leadership process (22), the leadership literature has placed a major focus on leadership position or leadership role occupancy, defined as whether a person occupies a leader position to supervise other individuals (21), and managing demands, referring to holding job positions that require management of other people, resources, and work tasks (23). We adopted these two job position–related leadership variables in the current research.

For GWAS analyses, we treated leadership position as a binary variable (leaders versus nonleaders) obtained from the UK SOC 2000 system. For the managing-demands variable, we linked the UK SOC 2000 job codes in the UKB data to the occupation codes from the O*NET database to construct a score indicating the levels of the managing demands by averaging ratings of nine items from the O*NET (24, 25) (*SI Appendix*, Fig. S1 and *Materials and Methods*). Phenotypic descriptions of the leadership position and the nine items of managing demands were listed in *SI Appendix*, Table S1.

In the analyses with the UKB data, we included 248,640 participants of European ancestry with 17.29% being leaders (42,998 cases) and 82.71% as nonleaders (205,642 controls; Table 1 and *SI Appendix*, Table S2 and Fig. S2). Among all the participants, 48.11% were males and the average age was 54.3 y, ranging from 39 to 71. A subset of 219,474 participants with available data on the managing demands phenotype was included in the discovery stage. The average managing demands score was 3.13, ranging from 0.95 to 5.12. Leadership position was moderately correlated with managing demands for the whole sample (Pearson correlation *r*: 0.63) or by sex (males: 0.66; females: 0.57; *SI Appendix*, Table S3). In general, those who held leadership positions, compared to their nonleader counterparts, had higher education qualifications (37.94% versus 34. 16% with college or university degree), higher household income (11.93% versus 4.75% with an annual income greater than 100,000 pounds), lower Townsend deprivation index (−1.98 versus −1.51), more likely owned accommodation lived in (94.6% versus 91.6%), had more vehicles in the household (66.43% versus 54.98% with two or more cars), and relied more on the car for commuting to the workplace (70.1% versus 64.9%; *SI Appendix*, Table S4).

**Table 1.** Summary of the leadership phenotypes in the UKB discovery sample

Leadership position	N	Leaders (%)	Nonleader (%)
All	248,640	42,998 (17.29)	205,642 (82.71)	
Male	119,618	27,066 (22.63)	92,552 (77.37)
Female	129,022	15,932 (12.35)	113,090 (87.65)
Managing demands		Mean	SD
All	219,474	3.13	0.93
Male	108,685	3.25	0.94
Female	110,789	3.01	0.88

The table shows information for individuals of European ancestry in UKB data included for GWAS analyses with valid phenotype and genetic data passed QC. Managing demands are measured by the score, with a high score reflecting a high level of managing demands.

We also performed replication analyses in three independent datasets: the UKB follow-up dataset (*n* = 22,875), the Add Health Wave IV dataset (*n* = 5,141), and the Wisconsin Longitudinal Study dataset (WLS; *n* = 5,899). The mean age (years) of participants was 60.4 in the UKB follow-up dataset (43.8% male), 28.4 in the Add Health dataset (46.8% males), and 68.6 in the WLS cohort (49.2% males; *SI Appendix*, Table S5).

### GWAS for Leadership Position and Managing Demands.

Following a prespecified analysis plan, we performed GWAS for leadership position and managing demands on 9,804,641 variants passed quality control (QC) with minor allele frequency (MAF) >1% in 248,640 White participants from UKB data. The sex-stratified GWAS were also performed. The quantile–quantile (Q–Q) plots showed little evidence of inflation in test statistics for leadership position (λ_GC_ = 1.05; *SI Appendix*, Fig. S3 and Table S6). For managing demands, there was moderate inflation of the test statistics (λ_GC_ ranging from 1.10 to 1.20). However, the estimated linkage disequilibrium score (LDSC) regression intercept (ranging from 1.02 to 1.05) suggests that the inflation was largely due to the presence of polygenic inheritance (26); thus, we did not perform a correction for λ_GC_.

**Fig. 1.**
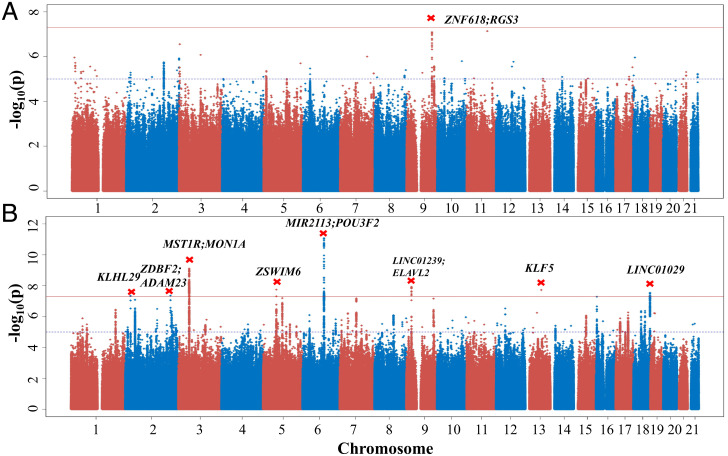
Manhattan plot of GWAS analysis for leadership traits in the UKB discovery sample. Results are shown for (*A*) leadership position (*n* = 42,998/205,642) and (*B*) leadership managing demands (*n* = 219,474). The *y*-axis represents −log_10_(*P* value) for association test with each phenotype, and the *x*-axis represents genomic position based on human genome build 37. The cross in red represents independent genome-wide significant association signals labeled by names of the genes or nearest genes. The horizontal red line indicates the significance level of *P* < 5.0 × 10^−8^. The horizontal blue dashed line indicates the suggestive significance level of *P* < 1.0 × 10^−5^.

Initially, we identified nine potential signals (*P* < 5 × 10^−8^) for the whole sample in the UKB discovery phase for the single-trait analysis (Fig. 1, Manhattan plots S4 and regional plots S5). The most significant signals were at *miR2113*/*POU3F2* on chromosome 6 for managing demands. We further assessed these signals in the independent replication samples. Three of the loci replicated at a nominal *P* value in the replication datasets (Wald test *P* = 0.0389 for rs4665237 at *KLHL29*, *P* = 0.0175 for rs1487441 at *miR2113*/*POU3F2*, and *P* = 0.0186 for rs76915478 at *KLF5*; *SI Appendix*, Table S7), despite the much smaller sample size (total sample size = 33,915 individuals). All top single-nucleotide polymorphisms (SNPs) except one retained genome-wide significance in the meta-analysis combining all datasets, with the effect sizes and directions largely concordant (heterogeneity *P* value >0.172). For sex-specific analyses, we identified three loci, among which one signal for managing demands at gene *NPAS2* on chromosome 2 in females remained significant across the discovery and replication samples. The nine independent SNPs showing genome-wide significance across all the datasets were reported in Table 2.

**Table 2.** Summary of top loci for leadership traits identified from UKB data

SNP	CHR	BP	A1/A2	Function	Locus	MAF	β (SE)	*P-discovery*	*P-meta*	*P-het*	*P-pleio*
Leadership position											
rs7035099	9	116568694	T/C	Intergenic	*ZNF618;RGS3*	0.42	−0.018 (0.008)	2.20 × 10^−8^	6.14 × 10^−9^	0.391	N.A.
Managing demands											
rs4665237	2	23900526	T/G	Intronic	*KLHL29*	0.47	0.015 (0.003)	4.60 × 10^−8^	5.22 × 10^−9^	0.683	0.066
rs9848497	3	49951316	T/C	Intergenic	*MST1R;MON1A*	0.48	0.017 (0.003)	8.90 × 10^−10^	2.21 × 10^−9^	0.330	0.058
rs7719676	5	60736949	A/G	Intronic	*ZSWIM6*	0.33	0.017 (0.003)	1.80 × 10^−8^	6.53 × 10^−9^	0.827	0.136
rs1487441	6	98553894	A/G	Intergenic	*MIR2113;POU3F2*	0.48	0.019 (0.003)	8.50 × 10^−12^	5.17 × 10^−13^	0.908	3.76 × 10^−4^
rs4977839	9	23355310	A/G	Intergenic	*LINC01239:ELAVL2*	0.42	0.016 (0.003)	1.20 × 10^−8^	1.20 × 10^−8^	0.664	0.167
rs76915478	13	73639505	A/G	Intronic	*KLF5*	0.08	−0.029 (0.005)	1.90 × 10^−8^	1.10 × 10^−9^	0.301	7.77 × 10^−3^
rs61532083	18	75891789	A/G	Intergenic	*LINC01029*	0.27	0.017 (0.003)	3.10 × 10^−8^	3.09 × 10^−8^	0.383	5.21 × 10^−3^
rs11541353*	2	101594191	T/C	Missense	*NPAS2*	0.18	−0.027 (0.005)	2.80 × 10^−8^	3.15 × 10^−8^	0.312	6.10 × 10^−3^

Lead variants shown were *P-meta* < 5 × 10^−8^ for leadership position and managing demands in individuals of European ancestry from the discovery UKB dataset, UKB follow-up cohorts, and the Add Health dataset. *P-discovery* is the *P* value from the discovery UKB dataset. For leadership position, the sample size for the UKB discovery: N = 42,998/205,642; All: *n* = 282,555. For managing demands, the sample size for the UKB discovery: N = 219,474; All: *n* = 250,423. The replication studies include the UKB follow-up cohort, Add Health study, and WLS. For the variant associated with managing demands, we assessed their pleiotropic association with the leadership across all datasets, reflected by the *P-pleio*. All significant variants from the UKB discovery samples were presented in *SI Appendix*, Table S7. CHR, chromosome; A1, effect allele; A2, reference allele; β, beta effect based on the effect allele A1. *P-het*, heterogeneity *P* value across discovery and replication samples.

*rs11541353 is the genome-wide significant variant for managing demands in female participants and remained significant in both discovery and replication samples.

For the top SNPs for managing demands, we tested whether they showed associations with leadership position in the discovery and replication samples. For the lead SNPs at eight loci, the alleles associated with the increased levels of managing demands were all positively associated with the leadership position (*SI Appendix*, Table S8). Three loci, *miR2113*/*POU3F2*, *LINC01029*, and *NPAS2,* exhibited significant pleiotropic associations for leadership position after Bonferroni correction (*P-pleio* < 6.25 × 10^−3^; Table 2).

We also conducted multitrait analyses on leadership position and managing demands using the multitrait analysis of GWAS (MTAG) method (27) with leadership position as the primary trait. Overall λ_GC_ was 1.05, consistent with a polygenic inheritance model for leadership phenotypes (Q–Q plots; *SI Appendix*, Fig. S6). By incorporating information from the managing demands, we noted that Z-score statistics were boosted for testing the association between variants and leadership-position phenotype. With the increased statistical power, MTAG-leadership results were employed in the heritability and genetic correlation analyses, along with the GWAS results from the single-trait analyses.

### Biological Functions of Top GWAS Loci.

The most significant association signal across all cohorts falls within chromosome 6q16.1, located in an intergenic region between *miR2113* and *POU3F2*, which contains dozens of putative fetal brain–specific enhancers. Intriguingly, the lead SNP rs1487441-A allele associated with increased managing demands (and a higher level of leadership position as well) in our study was previously reported to be associated with an increased risk for bipolar disorder (*P* = 2.58 × 10^−8^) (28). GWAS results of educational attainment and intelligence also pinpointed the same locus (19, 29), suggesting a common underlying biological mechanism for these phenotypes. Animal models showed that the top SNPs in linkage disequilibrium (LD) at 6q16.1 regulated *POU3F2*, a transcription factor that was involved in the neurogenesis, maturation, and migration of upper-layer cortical neurons (30). Consistently, another top locus in the intergenic region at *LINC01239/ELAVL2* on 9p21.3 in the present study has also been identified as a genome-wide significant locus for bipolar disorder (28). The top SNP rs4977839-A allele for a higher level of managing demands was also significantly associated with an increased risk for bipolar disorder (*P* = 6.90 × 10^−9^) (28).

The second most significant signal lies in the *KLF5* gene on chromosome 13q22.1. The *KLF5* gene encodes a member of the Kruppel-like factor subfamily of zinc-finger proteins, which is enriched in the motifs of transcription factors for schizophrenia (31). Additionally, another association signal at gene *ZSWIM6* located on chromosome 5q12.1 has been found as GWAS loci for schizophrenia. The lead SNP rs7719676-A allele for a higher level of managing demands in our study was significantly associated with decreased risk of schizophrenia (*P* = 1.22 × 10^−9^) (32). Zswim6 knockout mice had an alternation in striatal morphology and motor control (33), an important contributor to brain function.

For the leadership-position phenotype, the top signal is located in an intergenic region between *ZNF618* and *RGS3* on chromosome 9q32. The *ZNF618* gene encodes zinc-finger protein 618, and rgs3 is a GTPase-activating protein that inhibits G protein–mediated signal transduction. The rs7035099-C allele associated with leadership position was also associated with, although not reaching genome-wide significance, an increased level of risk tolerance (*P* = 4.91 × 10^−4^) (34) and extraversion (*P* = 9.55 × 10^−4^) (35).

For female-specific loci, the lead SNP rs11541353 is a missense variant in the *NPAS2* gene on chromosome 2q11.2 (*p. Ser471Leu*). The *NPAS2* gene codes neuronal PAS domain protein 2. It plays a key role in the acquisition of memory; such epistatic clock genes are potentially involved in the etiology of autistic disorder (36).

### Heritability and Genetic Correlations among the Leadership Traits across Sex.

Overall, common SNP heritability (SNP-*h*^2^) for the leadership-position trait was estimated to be in the range between 3 and 9% (95% CI, 2 to 11%) from GWAS summary statistics using the LDSC method for the whole sample and sex-specific samples (*SI Appendix*, Table S9). The estimates, ranging from 5 to 10% (95% CI, 3 to 11%), were similar to those using the BOLT-restricted maximum likelihood (BOLT-REML) method (https://data.broadinstitute.org/alkesgroup/BOLT-LMM/downloads). Overall, for leadership position and managing demands, the SNP-*h*^2^ estimates ranged from 3 to 8% and 4 to 10%, respectively. For MTAG-leadership, the SNP-*h*^2^ was estimated to range from 4 to 9%. Across sexes, there were substantial genetic correlations for MTAG-leadership, leadership position, and managing demands (*r_g_* ≥ 0.88; *SI Appendix*, Table S10). A partially shared genetic architecture was observed between leadership position and managing demands (*r_g_* = 0.57) as well as in males and females.

In sensitivity analyses, we estimated SNP-*h*^2^ for senior leadership position with 166,791 individuals (among them 3,834 senior leaders) in the UKB and Add Health datasets (*SI Appendix*, Table S11). We speculate that those holding senior leadership positions may have a higher heritability. As the sample size for the senior leader in each cohort was small, we performed a meta-analysis to combine GWAS summary statistics for senior leadership across the three datasets (Q–Q plot; *SI Appendix*, Fig. S7). The SNP-*h*^2^ for the whole sample was estimated at 7% (95% CI, 1 to 13%), slightly higher than that for leadership position (*SI Appendix*, Table S9); the 95% CIs were overlapped. Meanwhile, there was partially shared genetic architecture between the senior leadership position and leadership position (0.58, 95% CI, 0.27 to 0.90).

The SNP-*h*^2^ estimates for leadership traits were lower than those estimated from the twin studies (around 30%) (4, 6, 7), partially due to the contributions from the polygeny, nonadditive genetic effects, and rare and structural variants. It is also likely that the LDSC estimates could be downward to null. The SNP-*h*^2^ estimates for leadership position and managing demands are in line with those for other behavior traits (*SI Appendix*, Fig. S8), such as risk tolerance and extraversion, and slightly larger than agreeableness and subjective well-being but less than physical traits, metabolites such as high-density lipoprotein (HDL), low-density lipoprotein (LDL), body mass index (BMI), and intelligence.

### Association of Polygenic Score with Leadership Position.

To test whether the aggregate estimates of genetic effects are associated with leadership position, we constructed polygenic scores (PGSs) based on the MTAG-leadership GWAS summary statistics in the independent UKB follow-up dataset. The PGSs were significantly associated with the leadership position at varying threshold *P* values (cutoffs at 1, 0.05, and 1 × 10^−3^) and with the Lassosum approach (model fitting *P* for PGSs ranged from 3.27 ×10^−4^ to 0.011; *SI Appendix*, Table S12). Using PGSs generated at the threshold of 0.05 (see distribution in *SI Appendix*, Fig. S9), we categorized PGSs into five quantiles in the logistic regression to model leadership position, accounting for age, sex, and genotyping arrays. The top PGS quantiles (top 20th), as compared to the lowest 20th quantiles, were associated with an increased likelihood to hold a leadership position (odds ratio [OR] = 1.22, 95% CI, 1.08 to 1.36; *P* = 8.42 × 10^−4^) as well as a senior leadership position (OR = 1.33, 95% CI, 1.09 to 1.63; *P* = 4.76 × 10^−3^; *SI Appendix*, Table S13). PGSs accounted for a small amount of variance of leadership position, with an incremental R^2^ at 0.04%, on top of age and sex.

### Genetic Correlations between Leadership and Personal Traits.

Next, we assessed genetic correlations between leadership with 10 personal traits that have been shown to correlate with leadership, including intelligence, Big-Five personality traits, risk tolerance, height, educational attainment, and income, using summary statistics from previous GWAS (*Materials and Methods* and *SI Appendix*, Table S14). The significance level was set at a false discovery rate (FDR) <0.05 to account for multiple testing (37). Intelligence is one of the most well-studied psychological traits predictive of work and life achievements (2, 3), with leadership position as one of them (4, 38, 39). The Big-Five personality traits have been used as the overarching taxonomy to organize personality traits in leadership studies (40). Previous studies also demonstrated that, phenotypically, risk-taking (41, 42) and height were positively and significantly associated with leadership (43–45).

We found that the leadership position variable had significant genetic correlations with risk tolerance (*r_g_* = 0.40, 95% CI, 0.31 to 0.49), neuroticism (*r_g_* = −0.19, 95% CI, −0.26 to −0.11), intelligence (*r_g_* = 0.20, 95% CI, 0.11 to 0.30), and height (*r_g_* = 0.11, 95% CI, 0.05 to 0.17; *SI Appendix*, Table S15). Leadership position was genetically correlated with extraversion at nominal significance (*r_g_* = 0.51, 95% CI, 0.01 to 1.01). For MTAG-leadership, integrating information from the managing demands phenotype, the genetic correlation with intelligence substantially increased compared to analyses with leadership position variable alone (*r_g_* = 0.48, 95% CI, 0.40 to 0.56). The genetic correlation remained significant for other variables: risk tolerance (*r_g_* = 0.32, 95% CI, 0.25 to 0.40), neuroticism (*r_g_* = −0.25, 95% CI, −0.32 to −0.18), height (*r_g_* = 0.17, 95% CI, 0.12 to 0.22), and extraversion (*r_g_* = 0.49, 95% CI, 0.07 to 0.91). The genetic correlation for leadership position was higher with income than educational attainment (educational attainment: *r_g_* = 0.18, 95% CI, 0.11 to 0.24; income: *r_g_* = 0.53, 95% CI, 0.44 to 0.62); substantially stronger correlations were observed for MTAG-leadership in a similar pattern (educational attainment: *r_g_* = 0.58, 95% CI, 0.52 to 0.63; income: *r_g_* = 0.83, 95% CI, 0.75 to 0.90).

### Genetic Correlations between Leadership and Well-Being.

**Fig. 2.**
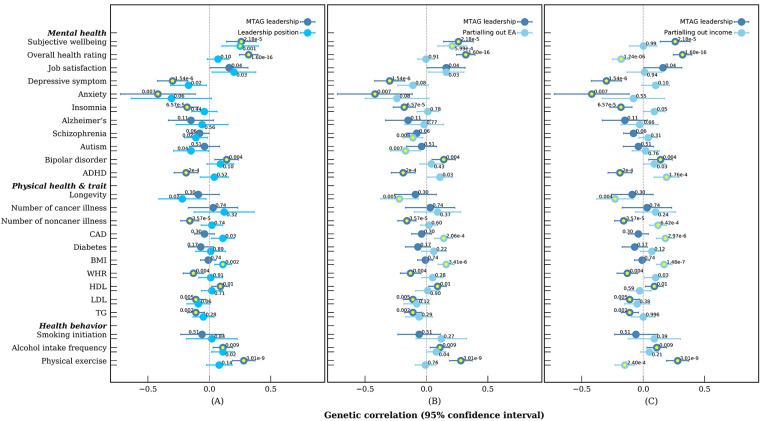
Genetic correlations of leadership position phenotypes with health indicators and health behaviors. (*A*) MTAG-leadership and leadership position. (*B*) MTAG-leadership before and after partialing out genetic variance related to educational attainment. (*C*) MTAG-leadership before and after partialing out genetic variance related to income. *P* values for the genetic correlations are reported above each dot. Horizontal bars represent 95% CIs. Yellow asterisks denote the genetic correlations at FDR < 0.05. WHR, waist–hip ratio; TG, triglycerides.

In the UKB data, we found positive genetic correlations of leadership position and MTAG-leadership with positive physical and mental health and well-being variables at large. For MTAG-leadership, there were positive genetic correlations with subjective well-being (*r_g_* = 0.26, 95% CI, 0.14 to 0.39; Fig. 2*A* and *SI Appendix*, Table S16), overall health rating (*r_g_* = 0.32, 95% CI, 0.25 to 0.40), HDL (*r_g_* = 0.09, 95% CI, 0.02 to 0.16), physical exercise (*r_g_* = 0.28, 95% CI, 0.19 to 0.37), and negative correlations with depression symptoms (*r_g_* = −0.30, 95% CI, −0.42 to −0.18), anxiety (*r_g_* = −0.42, 95% CI, −0.72 to −0.11), attention deficit hyperactivity disorder (ADHD) (*r_g_* = −0.19, 95% CI, −0.28 to −0.09), number of noncancer illness (*r_g_* = −0.16, 95% CI, −0.23 to −0.08), LDL(*r_g_* = −0.11, 95% CI, −0.19 to −0.03), and triglycerides (TG) (*r_g_* = −0.11, 95% CI, −0.18 to −0.04). Moreover, we also observed that leadership was positively and genetically correlated to bipolar disorder (*r_g_* = 0.14, 95% CI, 0.04 to 0.24) and alcohol intake frequency (*r_g_* = 0.11, 95% CI, 0.03 to 0.19), both being indicators of low levels of health and well-being. In general, the genetic correlation estimates with the variable leadership position alone were similar to, and sometimes lower than, those with MTAG-leadership. However, leadership position was significantly correlated with BMI (*r_g_* = 0.11, 95% CI, 0.04 to 0.18) and cardiovascular disease (CAD) (*r_g_* = 0.11, 95% CI, 0.01 to 0.20), while these two indices were not significant correlated with MTAG-leadership (*r_g_* = −0.01 and −0.04 for BMI and CAD, respectively).

Leadership position, as an indicator of SES pertaining to one’s occupational achievement, is associated with other SES measures such as educational attainment and income, each of which has shown genetic correlations with positive health indicators in most instances (45). Occupying leadership positions at work can be considered as a career achievement that may share similar advantages as other SES conditions on well-being. Thus, it is important to examine whether a leadership position holds a unique genetic association with well-being, after accounting for the genetic effects of other SES measures. The genetic correlations between MTAG-leadership (or leadership position) and well-being before and after removing genetic variance in common with the educational attainment or income were presented in Fig. 2 *B* and *C*, with exact estimates in *SI Appendix*, Table S16. Whereas the genetic correlations were substantially weakened and became nonsignificant with a lot of well-being indices (depressive symptoms, anxiety, insomnia, waist–hip ratio, DHL, LDL, and TG), the findings also reveal unique genetic correlations between some well-being variables with leadership position. Notably, the significant positive genetic correlation remained at a similar magnitude for subjective well-being after accounting for genetic influences related to educational attainment; the positive genetic correlations became negative for overall health rating and physical exercise, while the negative genetic correlations became positive for ADHD and number of noncancer illnesses after partialing out genetic influence related to income. The change of genetic correlations was mostly evident after partialing out the genetic variance of income, perhaps in part because the genetic variants associated with high income tend to be more strongly associated with well-being variables than the genetic variants for leadership position. Intriguingly, the negative genetic correlations between leadership position (or null for MTAG-leadership) and some well-being variables (longevity, CAD, and BMI) were strengthened and became significant after accounting for both educational attainment and income. The positive genetic correlations between leadership position and shortened longevity, CAD and BMI, were in contrast to the directions of genetic correlations observed for both educational attainment and income (18).

**Fig. 3.**
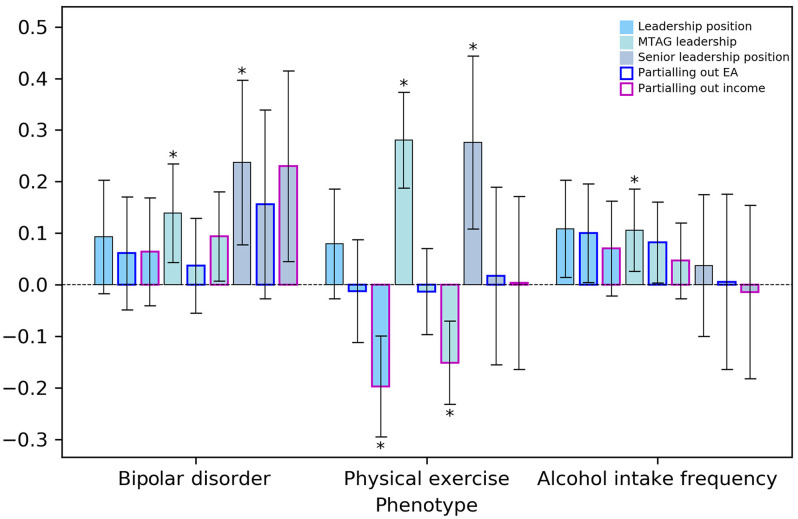
Genetic correlations of leadership traits with bipolar disorder, physical exercise, and alcohol use. Genetic correlation for the four leadership traits: MTAG-leadership, leadership position, managing demands, and senior leadership position. Vertical bars represent 95% CIs. Asterisks denote the genetic correlations at FDR < 0.05.

The genetic correlations between leadership position and an increased risk of bipolar disorder and alcohol intake frequency, as well as physical exercise, were further assessed for those holding senior leadership positions. Senior leaders had a larger genetic risk for bipolar disorder (*r_g_* = 0.24, 95% CI, 0.08 to 0.40; Fig. 3), about 2.5-fold of the correlation for the leadership position variable (*r_g_* = 0.09, 95% CI, −0.02 to 0.20) and 1.7-fold of MTAG-leadership (*r_g_* = 0.14, 95% CI, 0.04 to 0.24). The 95% CIs were wide for senior leadership likely due to the small sample size, whereas the trend suggested that the shared genetic factors predisposing to bipolar disorder could be potentially more pronounced for senior leaders. A strengthened positive genetic correlation was observed between senior leadership and physical exercise (*r_g_* = 0.28, 95% CI, 0.11 to 0.45). Such a pattern, however, was not observed for alcohol intake frequency (*r_g_* = 0.04, 95% CI, −0.10 to 0.18).

After partialing out the genetic variance related to other SES variables, the genetic correlations were substantially weakened and became nonsignificant with alcohol intake frequency. For MTAG-leadership, the genetic correlation was marginally significant with bipolar disorder after partialing out genetic influences related to income (*r_g_* = 0.09, 95% CI, 0.01 to 0.18) and changed to the opposite direction with physical exercise after accounting for genetic influences related to income (*r_g_* = −0.15, 95% CI, −0.07 to −0.23). For analyses with those holding senior leadership positions, the genetic correlation with bipolar disorder attenuated after partialing out genetic variance related to educational attainment (*r_g_* = 0.16, 95% CI, −0.03 to 0.34), whereas it remained at a similar magnitude, but marginally significant, after partialing out income (*r_g_* = 0.23, 95% CI, 0.05 to 0.41). The results suggest that bipolar disorder could link to common biological mechanisms underlying educational attainment and leadership but not income.

## Discussion

In the current research, we reported the most comprehensive GWAS on leadership and genetic correlations between leadership and personal traits, as well as one’s well-being variables, using large-scale datasets. First, we found evidence for the contribution of common genetic variation for leadership traits by identifying eight genome-wide significant loci across sexes and one in female participants. The top loci overlapped with GWAS hits for bipolar disorder and schizophrenia and were also related to personal traits such as intelligence, risk tolerance, and extraversion. PGS combining common variants had small but significant predictive value for the leadership traits. Second, we demonstrated, on the one hand, a consistent pattern of positive genetic correlations between leadership position and positive well-being indicators. On the other hand, leadership position had a positive genetic correlation with poor well-being indicators, including bipolar disorder and alcohol intake frequency. After partialing out the genetic variance in common with other SES measures, positive genetic correlations between leadership with the low health status (shortened longevity, CAD, and BMI), as well as the low levels of physical exercise, emerged. Our study demonstrated the polygenetic nature of leadership, the shared genetic basis between leadership traits and a broad range of well-being indicators, and the unique associations with well-being after accounting for genetic influences related to other SES measures.

Leadership has been an essential and classic topic in genetic research perhaps dating back to the early 19th century, when modern human genetics as a scientific area was first established (46). Modern genetics research on leadership appeared much later using the classic twin approach (6, 7). Our study advanced this line of inquiry by providing results from a whole-genome exploration of leadership and unraveling genetic correlations between leadership and various well-being variables. Our findings corroborated with previous twin and candidate gene studies on significant heredity of leadership and pinpointed some associated genes. Our research has revealed intriguing results. For example, the lead SNP rs1487441-A allele at loci *miR-2113/POUSF2* and the rs4977839-A allele at *LINC01239/ELAVL2* associated with leadership position and a high level of managing demands were also found to be associated with an increased risk for bipolar disorder (19, 29). The findings corroborated with the positive genetic correlation between bipolar disorder and leadership traits found using the whole-genome data. Our GWAS results suggest that the leadership phenotypes are significantly heritable traits but are highly polygenic. Similar to most personality traits (i.e., risk tolerance, extraversion, and conscientiousness) (29, 34, 47), the heredity estimates from our GWAS accounted for a small but comparable fraction of genetic variance (<10%). We speculated that thousands of variants with small effects spreading across the whole genome contribute to individual differences in predisposition to leadership. Such phenomena may pose tremendous challenges to precisely estimate the true effect sizes of variants in the discovery dataset. Despite the limitation of using linear additive effects of common SNPs in heritability and PGS calculation, our results suggest the polygenity and complexity of genetic structure underlying leadership phenotypes.

We tested the genetic correlations between leadership position and a set of personal traits that are predictive of leadership. At the phenotypic level, leadership has been reported to be positively related to intelligence, extraversion, risk tolerance, education, income, and height and negatively related to neuroticism (38, 40, 41, 43). Consistent with phenotypical studies, intelligence, extraversion, risk tolerance, neuroticism, and height had significant genetic associations with leadership in our research. The signs (positive or negative) of the phenotypic and genetic correlations between leadership position and such personal traits were consistent, which points to the possibility that shared genetic architecture may underpin the corresponding phenotypical relationships. Genetic influences on leadership may be carried through such personal traits (4, 38, 39).

Previous research has provided contrasting views and mixed evidence on whether holding a leadership role is beneficial or detrimental to one’s well-being. Some argued that leaders may shoulder high job demands, which may erode their health and well-being, while others reason that leaders’ experiencing a strong sense of control over their work may bring health benefits to them. These two offsetting mechanisms may lead to varied direction and strength in the relationship between leadership and well-being, depending on the context and types of well-being outcome (15). In the current study, most genetic correlations between leadership and positive well-being indicators were positive, similar to findings for intelligence (47), education achievement (48), job attainment (49), and income (18).

After partitioning out the genetic effects related to education or income, a lot of those significant genetic correlations became less significant, suggesting that leadership position has substantial genetic overlap with education and income in their positive relationships with well-being. However, there were instances of divergence indicating unique genetic associations between well-being and leadership position. This means that genetic influences associated with leadership position may be detrimental to well-being, the results of which are different from those for educational attainment and income. Such evidence at the genomic level provides support for findings on contrasting mechanisms for the phenotypic relationship between leadership and well-being (15). Particularly, after partitioning out effects of educational attainment or income, holding leadership positions was genetically and positively associated with higher BMI, increased risk for CAD, and further reduced longevity. The high psychological demands embedded in holding leadership positions—a form of chronic stressors—might play a role because they tend to stimulate psychobiological stress responses, including changes in fat metabolism and cardiovascular function, which are detrimental to health in the long run (50).

We observed positive genetic correlations between leadership position and bipolar disorder before partitioning out genetic influences related to income or education achievement, and such genetic correlations appeared particularly more pronounced in senior leaders. Furthermore, our research pinpointed two top loci—miR-2113/*POUSF2* and *LINC01239 ELAVL2*—that were associated with increased bipolar-disorder risk and higher intelligence, suggesting a common underlying biological mechanism of these traits. Such findings may explain the attenuated genetic correlation for bipolar disorder after partitioning out genetic influences related to educational attainment but not income. Income is a more distal phenotype from DNA than education; thus, it is likely a more proximal predictor of health and well-being. Phenotypic research has portrayed bipolar disorder as a mixed blessing for leadership. Researchers reported a positive association between bipolar disorder and superior leadership qualities in a Swedish population study (16). Bipolar disorder has also been found to be positively associated with childhood intelligence quotient (IQ) (51), creativity (52), and entrepreneurship (53). Research also reported positive genetic associations between bipolar disorder, intelligence, (54) and education (55). The elevated behavioral activation system sensitivity may be an explanation for genetic associations between bipolar disorder with these traits and achievements (56). Yet prior research has also reported that bipolar disorder is dysfunctional in terms of affecting one’s job performance (57). Future research should examine causal associations among these traits more comprehensively.

We also observed mixed relationships between leadership position and health behaviors. Leadership position had a positive genetic correlation with physical exercise, which is beneficial to health and well-being. Partialing out genetic influences related to income, the positive genetic relationship between leadership position and physical exercise turned negative. This might have to do with heightened psychological demands for those holding leadership positions, which might be partially genetic and uniquely related to leadership. High psychological demands may increase leaders’ stress levels and reduce their time to engage in physical exercise. Leadership position was genetically correlated with a high level of alcohol intake frequency—an unhealthy behavior. Alcohol intake frequency was reported to be genetically positively correlated with other SES indices, including education, income, and the reversed Townsend deprivation index (58). Our results corroborated these findings and suggest shared genetic mechanisms for leadership position and other SES indices in their relationships with alcohol intake frequency. These results may also partially explain the mixed findings for the phenotypic relationship between leadership and health outcomes.

The study has several limitations. First, the nature of leadership is complex and multifaceted. Our measures of leadership position and managing demands were extracted from occupational databases and only included executive, managerial, and supervisory roles and tasks in work settings. Although consistent with the literature on leadership (6, 7), our measures are not able to capture the whole spectrum of leadership, such as leadership effectiveness (40), leadership styles (59), and leadership roles in other social settings. Future genomic studies should examine other different leadership measures. Second, to build up a fuller biological foundation of leadership beyond genetics, we still need other important physiological mechanisms and to consider environmental influences. Hormones, such as testosterone, oxytocin, and serotonin, may be related to leadership (60, 61). Some preliminary evidence suggests that certain brain structures and functions are relevant to leadership (52, 53). Future genomic studies need to reveal such physiological pathways from genetics to leadership and the role of environments beyond psychological and physical traits explored in the current study.

Our findings offer insights into the biological underpinnings of leadership, revealing top loci overlapping with those for mental health traits and the pervasive polygenity of work-related variables. We also found evidence for the shared and unique genetic correlations between leadership traits and well-being after accounting for other SES measures.

While we encourage future research to further tackle the role of genetics in shaping work-related outcomes (e.g., leadership, work achievements, and entrepreneurship), findings on significant influences of specific genetic variants should not be explained as suggesting genetic determinism. As research on social influences, significant genetic influences suggest only that such influences are probabilistic, not deterministic. Environmental influences play important roles in mediating or moderating genetic influences. A more complete understanding of human behaviors should take various forms of interplay between nature and nurture into consideration.

## Materials and Methods

### Study Samples.

UKB data are from a population-based cohort study in the United Kingdom, which involves above 500,000 participants aged 40 y or older during their recruitment between 2006 and 2010 (62, 63). Participants provided their occupation information through interviews during their first visits to the UKB centers (64, 65), which were coded in the form of the four-digit UK SOC version 2000 (Field identifier: 132). In this study, we used data from 248,640 Caucasian individuals who answered the questions on occupation information and whose genotype data passed QC (*SI Appendix*, Fig. S1).

Replication samples included participants from the UKB follow-up cohort (*n* = 22,875), the Add Health Wave IV cohort (66) (*n* = 5,141), and the WLS cohort (67) (*n* = 5,899; *SI Appendix*, Fig. S10–S12 and Table S5). The details of replication cohorts were presented in *SI Appendix*, *Supplementary Notes*.

### Phenotype Definitions and Measures.

The phenotypes of interest are leadership position and managing demands (*SI Appendix*, Table S1). Leadership position refers to whether one person holds a supervisory position (21), which is a widely used indicator of leadership (38, 40). Previous leadership research has assessed leadership position according to whether participants occupy supervisory roles (6, 15, 68). Accordingly, in the UKB sample, we measured the leadership position based on whether the occupations require managing subordinates. The measure of leadership position was derived from participants’ occupational codes according to the UK SOC version 2000 system. In this classification system, there are three different skill levels for managerial occupations: senior leaders (e.g., directors and chief executives of major organizations), other corporate managers (e.g., marketing and sales managers), and managers and proprietors in agriculture and services (e.g., service managers). We coded participants with these occupations that required managing subordinates as leaders (1) and others as nonleaders (0). In the sensitivity analyses, we also performed GWAS for senior leaders (coded as 1) and nonleaders (0).

Leadership is also reflected in significant and unique job demands—managing other people, tasks, and resources (23, 69). The UK SOC version 2000 occupation codes from the UKB data were linked through a crosswalk to the US SOC version 2000 to extract further occupational characteristic information from the US O*NET database (70). Among 502,538 participants, we were able to match job titles for 274,223 individuals, while 192,010 did not provide SOC codes and 36,305 participants had SOC 2000 codes but without sufficient information that could be used in matching to the O*NET system (*SI Appendix*, Fig. S1). Managing demands were measured with nine items on typical managerial and supervisory tasks (24). These items were selected from the section of generalized work activities from the O*NET questionnaires. A sample item is “What level of guiding, directing, and motivating subordinates is needed to perform your current job?” All the items used a seven-point scale indicating the levels of the demands. The internal consistency reliability coefficient (Cronbach’s α) was 0.96. The measure of managing demands was presented as the average score across these nine items, with a higher score implying a higher level of demands.

For the Add Health Wave IV cohort, participants responded to the question “Thinking about your official job duties, which of the following statements best describes your supervisory responsibilities at your (current/most recent) primary job?” Response options ranged from 1 = “I supervise or supervised other employees,” 2 = “I supervise other employees, some of whom supervise others,” and 3 = “I do or did not supervise anyone.” We constructed the variable of the leadership position by assigning 1 to participants who selected the first and second options and 0 to those who chose the third option. In the sensitivity analyses, we selected those who chose 2 as those who hold senior leadership positions. After linking the occupational codes reported by participants to the O*NET database to extract leadership managing demands, we included 4,696 individuals in GWAS for managing demands.

For the WLS cohort, participants were asked three questions about their leadership position. The three items at each wave were “Do you have authority to hire and fire others at current/last job?” (1 = Yes, 0 = No), “Can you influence or set the rate of pay received by others at current/last job?” (1 = Yes, 0 = No), and “Do you supervise the work of others, that is, what they produce and how much at current/last job?” (1 = Yes, 0 = No). Given that participants were randomly selected to answer these questions at each wave, we used the average leadership position score across three items reported at their respective last wave (*n* = 5,899). Thus, the leadership position variable represents levels of managerial authority, influence, and responsibility. In addition, the US Census 1970 and 1990 job codes from WLS data were linked through crosswalks to the US O*NET-SOC version 2010 to extract the phenotype of managing demands from the US O*NET database. The WLS replication sample included 5,120 participants for managing demands.

In O*NET questionnaires, there is another item on leadership requirements (“How important is leadership to the performance of your current job?”). The phenotypical and genetic correlations between this item and managing demands were 0.74 and 0.97 in the UKB data. A separate GWAS analysis on this item generated similar results to those of managing demands. As this item was largely overlapping with managing demands, we thus dropped it from further analyses.

### Genotyping and Imputation.

We used imputation genotypes released by UKB (bgen files; imputed data version 3, released March 2018), which includes the full set of genotypes imputed on the Haplotype Reference Consortium (HRC) data, UK10K, and 1000 Genomes phase-3 reference panels. The QC and imputation were done by UKB and have been described elsewhere (62). A European subset was identified by projecting the UKB participants onto the 1000 Genomes Project principal components coordination. We excluded genetic variants with MAF <1% and poorly imputed markers (IMPUTE info < 0.3), resulting in 9,804,641 autosomal variants imputed or genotyped on 408,344 individuals of European ancestry. Among them, 248,640 individuals with the leadership-position phenotype were included in the analyses; 219,474 individuals were included for GWAS on managing demands. For the UKB follow-up dataset, the genotyping, imputation, and filtering procedure was similar to the one described for the UKB discovery, resulting in 22,875 individuals of European ancestry.

For the Add Health cohort, the genotypes were imputed on the HRC, with QCs detailed in ref. 71. A total of 7,508,602 genetic variants were included with MAF>1% and IMPUTE Info ≥0.3. Analyses were limited to 5,141 individuals of European ancestry, and cryptically related individuals were dropped from analyses. For the WLS cohort, the genotypes were imputed using the 1000 Genomes Phase-3 dataset. Replication analyses included 5,899 individuals of unrelated European participants. The QC and imputation for datasets used in this study were described in *SI Appendix*, *Supplementary Notes*.

### Genome-Wide Association Analyses.

We assumed an additive genetic model in which the dosage of each SNP was a continuous variable ranging from 0 to 2 for the effect allele. For UKB GWAS, a linear mixed model accounting for genetic relatedness was conducted to determine its association with the phenotype. The analyses were conducted with the software BOLT-LMM version 2.3.2 (https://alkesgroup.broadinstitute.org/BOLT-LMM/downloads) (72). The association analyses were adjusted for age, sex, genotyping array, and top 20 principal components (PCs). The GWASs were also done separately by sex.

Independent significant variants and their surrounding genomic loci were identified using LD clumping in PLINK version 1.07 (https://zzz.bwh.harvard.edu/plink/download.shtml). The LD structure was estimated from the European panels in the 1000 Genomes Project of phase 3 as the reference population. The index lead SNP was identified (*P* < 5 × 10^−8^), independent from other variants at each locus (*r*^2^ < 0.01). A locus was defined by an index SNP with the region flanking 500 kb on both sides. The coordinates and variant identifiers were reported on the National Center for Biotechnology Information B37 (hg19) genome build. The functional annotation and gene mapping were performed using ANNOVAR (version 2018Apr16), including types of intronic, exonic, intergenic, 5′-UTR, 3′-UTR, etc. (73). Regional plots of each identified locus were made by LocusZoom (http://locuszoom.org/), and the 1000 Genomes data of the European population was used to estimate LD information.

### Replication Analyses.

Genome-wide significant SNPs were evaluated in the UKB follow-up dataset, the Add Health dataset, and the WLS cohort. The detailed information for association analyses in the replication datasets is presented in *SI Appendix*, *Supplementary Notes*. We meta-analyzed results from both discovery and replication samples using the inverse-variance weighted fixed-effects model with METAL software (https://genome.sph.umich.edu/wiki/METAL). For the binary trait of leadership position, the coefficients obtained from the linear mixed model in the UKB discovery samples were on the standardized scale. Therefore, we transformed these coefficients to make them comparable with the observations in all the samples. We rescaled the beta coefficients with the following formula: β_s_ = β/k*(1−β), where *k* is the prevalence of leaders in the UKB; the ORs were calculated accordingly using the scaled beta coefficient β_s_.

### MTAG.

To combine the GWAS summary statistics for the two leadership variables, we applied the MTAG approach (27). MTAG is a method for joint analyses of summary statistics from GWASs of correlated traits, whereas the effect estimates for each trait can be improved by appropriately incorporating information contained in the GWAS estimates for the other traits. Here, we treated the phenotype, leadership position, as our primary trait; thus, MTAG returned association meta-analyses results for leadership position enriched by the results from the other leadership phenotype, managing demands. We labeled it as MTAG-leadership in this paper.

### Common SNP Heritability.

We used the software LDSC version 1.0.1 (https://github.com/bulik/ldsc) with GWAS summary statistics to estimate common SNP*-h*^2^ for three phenotypes: leadership position, managing demands, and MTAG-leadership for the whole sample and subsamples by sex in the UKB discovery data (26). We used LD-score regression to estimate the proportion of variance in liability to leadership traits that could be explained by the aggregated effect of the SNPs. Of 9,804,641 variants from GWAS summary-level data, we included SNPs presented in the European panels in the 1000 Genomes Project, with the exclusion of the major histocompatibility complex (MHC) region on chromosome 6. SNPs with INFO <0.8 were further removed, resulting in 1,174,163 SNPs for the LDSC regression analyses. We also applied the variance components analysis (BOLT-REML) method to estimate the genetic variance component of leadership traits (leadership position and managing demands) using individual-level autosomal genotype data of the UKB discovery sample. The analysis included 220,624 unrelated European samples in the UKB discovery phase using the software BOLT-LMM version 2.3.2 (https://alkesgroup.broadinstitute.org/BOLT-LMM/downloads). In the sensitivity analyses, we calculated SNP*-h*^2^ estimation of senior leadership phenotype using GWAS data in the UKB discovery sample with LDSC and BOLT-LMM approaches. We did not estimate SNP*-h*^2^ by sex due to the small sample size. To increase effective sample size, we also estimated heritability for senior leadership using meta-analysis results combining the results with the Add Health sample (*SI Appendix*, *Supplementary Notes*).

For the binary trait of leadership position, the estimated heritability should be transformed to the liability scale using the transformation derived by Lee et al. (74). As the exact prevalence is unknown, we assumed the percentage of leadership position in the UKB sample under current analysis is equal to the population prevalence (leadership position: 17.29%; male participants: 22.63%; female participants: 12.35%; senior leader: 1.59%), similar to calculating SNP*-h*^2^ on the liability scale for UKB dichotomous traits (http://www.nealelab.is/uk-biobank). For MTAG-leadership, we used the same percentage as for the leadership position. For the senior leadership position testing statistics from the meta-analysis across UKB and the Add Health cohorts, the prevalence is the weighted average percentage in UKB and the Add Health dataset (2.30% for senior leadership position).

### Genetic Correlations of Leadership Traits with Well-Being and Other Traits.

We first computed the genetic correlation between leadership position and managing demands, for the overall sample and subsamples by sex, using the GWAS summary statistics from the UKB discovery sample. We adopted the bivariate LDSC method by regressing the product of testing statistics (z-statistics) from each phenotype against the LD scores (75).

We assessed the genetic correlations with 32 personal traits and well-being variables using summary statistics from GWASs of European ancestry, with the detailed information of sample sizes, phenotypes, and GWAS summary data resources presented in *SI Appendix*, Table S14. We used summary statistics from the previously published large-scale GWAS or GWAS results from UKB data, for instance, subjective well-being (76), overall health rating (77), job satisfaction (UKB data field 4537), depressive symptoms (76), neuroticism (78), longevity (79), number of cancer illnesses (UKB data field 34), number of noncancer illnesses (UKB data field 135), smoking initiation (80), BMI (5), height (5), intelligence (29), etc. The bivariate LD-score regression was applied for genetic correlation analyses (75). Note that for the illustration purpose, in our results, we recoded job satisfaction and overall health rating so that higher scores indicate higher job satisfaction and overall health by flipping the sign of beta effects from original GWAS results.

We used two sets of summary statistics for leadership traits: 1) single-trait GWAS on the leadership position and 2) MTAG-leadership by combining the association statistics for the leadership position and managing demands using the MTAG method. We excluded variants (INFO < 0.8 and MAF < 0.01) and merged SNPs to the HapMap3 European panel; the MHC region on chromosome 6 was removed. Bivariate LDSC regression was applied for genetic correlations between each pair of leadership and other traits (75). As we estimated genetic correlations with a series of traits, we applied Benjamini–Hochberg FDR correlation for statistical significance when the FDR was less than 5% (37). In the sensitivity analysis, we also performed genetic correlation analyses using GWAS results of senior leadership and managing demands for bipolar disorder, alcohol use, and physical exercise.

To estimate the genetic correlations after partialing out the genetic variance of other socioeconomic variables (educational attainment and income), we used the Genomic SEM (81). For each pair of leadership and well-being phenotypes, we fitted the Genomic structure equation modeling (SEM) model including three traits: leadership position trait (X), well-being phenotype (Y), and education or income (Z). In the path diagram, there was a bidirectional arrow between two traits of X and Y and directional arrows from Z to X and Z to Y. The genetic effect of Z was regressed out from the variance of X and Y, affecting the genetic correlation. The genetic covariance matrix of X, Y, and Z was produced by the LDSC method implemented in Genomic SEM. The process was repeated for each well-being outcome.

### PGS Analyses.

A PGS estimates the cumulative effects of thousands of genetic variants identified from GWAS, including many with small effects. Using the GWAS results from MTAG-leadership, we generated PGSs in the UKB follow-up sample. The PGSs were constructed in PRSice (https://www.prsice.info/quick_start/), a method shown to have decent prediction accuracy involving LD pruning followed by *P-*value thresholding (82). Variants were selected as meeting a series of increasingly stringent *P*-value thresholds: *P* < 1, *P* < 0.05, *P* < 1 × 10^−3^, *P* < 1 × 10^−5^, and *P* < 1 × 10^−7^). Independent lead variants associated with the phenotype were identified by the “clumping and thresholding” approach, removing those within 250 kb and in linkage disequilibrium *r*^2^ ≥ 0.1 with the lead variant in the region. An individual’s PGS is a weighted sum of the genotypes across all independent variants. The weighting factor is the estimated additive effect size, beta coefficient, at each variant from the MTAG-leadership summary statistics. Prediction accuracy was based on an ordinary least squares regression of the leadership position on the PGS and a set of standard covariates, including age, sex, and the top genetic PCs. The McFadden pseudo-R^2^ for PGS was calculated as the incremental variance for leadership variables (i.e., the R^2^ of the model including PGSs and covariates minus the R^2^ of the model including only covariates). To evaluate the genetic effect on a leadership position for subjects in the highest quantiles of PGS, we performed the logistic regression for the leadership position variable, regressing on five quantiles of PGS, sex, age, and genetic array. Top genetic PCs were not included in the final model, as none of them was significant (*P* > 0.05). In sensitivity analyses, we applied Lassosum (https://github.com/tshmak/lassosum) to create PGS, with tuning parameters for the penalized regression across all the variants optimized in the testing samples (83).

## Supplementary Material

Supplementary File

## Data Availability

The UKB GWAS summary statistics for leadership position and managing demands can be downloaded from https://nusjobgmdata-public.s3.ap-southeast-1.amazonaws.com/ukb_leaderposition_all.txt.gz or https://nusjobgmdata-public.s3.ap-southeast-1.amazonaws.com/ukb_managing.txt.gz, respectively. All other data are included in the article and/or supporting information.
