# Supplementary material for: Genetics, leadership position, and well-being: An investigation with a large-scale GWAS
Source: Proc Natl Acad Sci U S A. 2022 Mar 14;119(12):e2114271119. doi: 10.1073/pnas.2114271119 (PMC8944770; doi:10.1073/pnas.2114271119)
Supplement: Supplementary File [file pnas.2114271119.sapp.pdf]

Genetics, Leadership Position, and Well-Being: An Investigation with a Large-Scale GWAS

Zhaoli Song, Wen-Dong Li, Xuye Jin, Junbiao Ying, Xin Zhang, Ying Song, Hengtong Li, Qiao Fan

Supplementary Materials and Methods

Supplementary Notes

1. Replication studies
2. Genotyping and imputation
3. Genetic association analysis in replication samples
4. Sensitivity analysis

Supplementary Figures

Supplementary Figure 1. Data preparation for the U.K. Biobank discovery sample.
Supplementary Figure 2. Distribution of leadership position and managing demands by sex.
Supplementary Figure 3. Quantile-quantile plots with genomic control inflation factor lambda λ_G.
Supplementary Figure 4. Manhattan plot of GWAS analysis for (a) leadership position (female); (b) leadership position (male); (c) managing demands (female); (d) managing demands (male).
Supplementary Figure 5. Regional plots for leadership position and managing demands.
Supplementary Figure 6. Quantile-quantile plots for MTAG-leadership.
Supplementary Figure 7. Quantile-quantile plot of GWAS meta-analysis results for senior leadership across UKB and the Add Health samples.
Supplementary Figure 8. SNP-h^2 heritability estimates for leadership position phenotypes, compared with a variety of other traits.
Supplementary Figure 9. Distribution of PGS constructed in the UKB follow-up cohort.
Supplementary Figure 10. Data preparation for the U.K. Biobank follow-up sample
Supplementary Figure 11. Data preparation for the Add Health Wave IV data.
Supplementary Figure 12. Data preparation for the Add Health Wave IV data

Supplementary Tables

Supplementary Table 1. Phenotypic description of leadership position and detailed items of managing demands
Supplementary Table 2. Summary of O*NET linked phenotype scores of managing demands in the U.K. Biobank discovery data
Supplementary Table 3. Phenotypic correlation between leadership position and managing demands
Supplementary Table 4. Summary of personal information in the U.K. Biobank discovery data
Supplementary Table 5. Sample size and demographic distribution in replication samples
Supplementary Table 6. Genomic control λ for GWAS on leadership traits in UK Biobank data
Supplementary Table 7. GWAS top variants for leadership position and managing demands phenotypes and replication
Supplementary Table 8. Top variants identified for managing demands and cross-trait association analysis on leadership position.
Supplementary Table 9. Summary of common SNP heritability estimations for leadership phenotypes from GWAS results in UKB data
Supplementary Table 10. Genetic correlation for leadership phenotypes across sexes in the UKB sample
Supplementary Table 11. Distribution of senior leadership position, and genomic control λ for GWAS results in three datasets
Supplementary Table 12. Constructing PGS for leadership in UKB follow-up cohort
Supplementary Table 13. Associations between PGS and leadership, or senior leadership position
Supplementary Table 14. Sources and phenotype description for GWAS results used in the genetic correlation analysis.
Supplementary Table 15. Genetic correlation between leadership position and personal traits, and social status measures
Supplementary Table 16. Genetic correlation between leadership position and health status.

1. Replication studies

The UKB follow-up participants were not included in the discovery UKB data. Between June to September 2015, about 102,000 UKB participants completed an online follow-up assessment on their employment history. We selected those who had past employment information but were not included in the discovery phase analysis because of lacking baseline job information. The replication sample comprised of 22,875 unrelated Caucasian individuals with valid job information of their last job, and genotype data (**Figure S10**).

The Add Health study is a U.S. multi-wave longitudinal study of adolescents (*1*). For the analyses of the Add Health Wave IV survey data that was collected between 2007 to 2009, we included 5,141 unrelated Caucasian participants with valid occupation information and genotype data (**Figure S11)**.

The Wisconsin Longitudinal Study (WLS) is a large U.S. multi-wave longitudinal study of a random sample of men and women who graduated from Wisconsin high schools in 1957 and of their randomly selected siblings (*2*). Six rounds of data collection were conducted from 1957 to 2011. The occupation information was collected in a random sample in the last four rounds: 1975-1977, 1992-1993, 2003-2004, and 2011. In this study, we extracted the leadership position and derived the managing demands phenotype from the last wave of each participant. The replication sample consists of 5,899 unrelated Caucasian participants with valid job and phenotype data (**Figure S12)**.

2. Genotyping and imputation

UK Biobank

We used imputation genotypes released by UK Biobank (bgen files; imputed data v3 – released March 2018). Samples were genotyped on the two customized SNP arrays (the UK BiLEVE Axion array and the UK Biobank Axiom array). The quality control and imputation were done by UK Biobank (*3*). Briefly, genotyped variants were filtered based on batch effects, plate effects, departures from HWE, genotype platform, and discordance across control replicates. Participants were excluded based on missing

rates larger than 5%, inconsistencies in reported versus genetic sex, and excessive heterozygosity based on a set of 605,876 high-quality autosomal markers. Genotypes were phased and the imputation was performed using IMPUTE4 with the Haplotype Reference Consortium (HRC) data, UK10K and 1000 Genomes Phase 3 dataset used as the reference set. We excluded genetic variants with MAF < 1%, and poorly imputed markers (IMPUTE info < 0.3), resulting in 9,804,641 autosomal variants imputed or genotyped on 408,344 individuals of European ancestry. Among them, 248,640 individuals with leadership position phenotype were included in the analyses; 219,474 individuals were included for GWAS on managing demands. For the UKB follow-up dataset, the genotyping, imputation, and filtering procedures were similar to the one described above for the UKB discovery, resulting in 22,875 individuals of European ancestry.

Add Health cohort

The sample of multiple ancestries were genotyped using Illumina's Human Omni1-Quad-BeadChip (N=9,947). In this study, analyses were limited to individuals of European-ancestry and cryptically related individuals, and ancestry outliers were dropped from analyses. Add Health study applied a stringent QC on genotyped data, variants with a per-variant missing call rate > 2%, MAF < 1% and Hardy-Weinberg Equilibrium $P < 1 \times 10^{-4}$ were excluded (*4*). In the individual-level filters, only participants of genetically ascertained European ethnicity were ascertained through the protocol developed for the association analyses of the GWAS & Sequencing Consortium of Alcohol and Nicotine use (GSCAN). Individuals with per-sample missing call rate > 5% and excessive-high or low heterozygosity were removed (F-statistics lower than -0.3 or higher than 0.3). Finally, an Identity-By-State (IBS) binomial test was conducted to filter out ancestral outliers using a threshold of 0.05 (https://addhealth.cpc.unc.edu/wp-content/uploads/docs/ user_guides/). After imputing the genetic data to the Haplotype Reference Consortium panel (HRCr1.1) 2016 using the Michigan Imputation Server, only HapMap3 variants were included, which are well imputed and provide good coverage of common variation across the genome. PCA analyses were conducted in GCTA v.1.92.4beta (*5*). To remove poor quality variants and likely false-significant associations, we excluded variants at imputed quality $R^2 <$ 0.3 and MAF < 1%, which resulted in 6,049,177 autosomal genetic variants.

WLS cohort

WLS participants were genotyped on the Illumina OmniExpress array, human Omni express-24-v1-1 annotation version A, designed to human genome hg19. 12.65% of the SNP assays attempted including uninformative redundant, monomorphic SNPs, and those with MAF < 1% were removed. IMPUTE2 software was used to perform genotype imputation in the WLS, using 1000 Genomes Project phase 3 reference panel. Participants with a missing call rate > 2% were removed. Therefore, the imputation included all 9,012 unique genotyped participants posted to dbGaP. Detailed QC and imputation report is at (https://www.ssc.wisc.edu/wlsresearch/documentation/GWAS/Herd_1000G_IMPUTE2report.pdf). In our replication study, we included 5,899 unrelated participants of Caucasian ancestry.

3. Genetic association analyses in replication samples

UKB follow-up data

The association analyses were conducted using linear or logistic regression, for management demands and leadership position respectively, in unrelated samples with PLINK2, adjusting for age, sex, genotyping array, and the top 20 principal components of the genetic data. Variants with MAF < 1% and with IMPUTE info <0.3 were removed from the analysis.

Add Health cohort

The association analysis was conducted using linear or logistic regression, for managing demands and leadership position respectively, in unrelated samples with PLINK2, adjusting for age, sex, and the top 4 principal components of the genetic data. Variants with MAF < 1% and IMPUTE info <0.3 were removed from the analyses.

WLS cohort

We perform association on 12 index SNPs for leadership position and managing demands in 5,899 unrelated participants of Caucasian ancestry. The linear regression model was performed using PLINK 2.0 with adjustment for covariance sex, age, and top 5 principal components.

4. Sensitivity analyses

The logistic regression analyses were conducted using PLINK2 in unrelated samples, adjusting for age, sex, genotyping array if possible, and top 20 principal components for UKB data (top 4 principal components for Add Health dataset). If the logistic regression fails to converge, the program will apply Firth regression instead automatedly. Variants with MAF < 1% and variants with IMPUTE info <0.3 were removed from the analyses.

To boost the power by increasing the effective sample size for heritability and genetic correlation analyses, we performed GWAS for the senior leadership position phenotype in each cohort: UKB discovery dataset, UKB follow-up cohort, and the Add Health study. We conducted the fix-effect meta-analyses implemented in Multi Trait Analysis of GWAS (MTAG) method (*6*). When the GWAS estimates are for the same trait in the non-overlapping samples, MATG method is the same for the inverse-variance weighted meta-analyses.

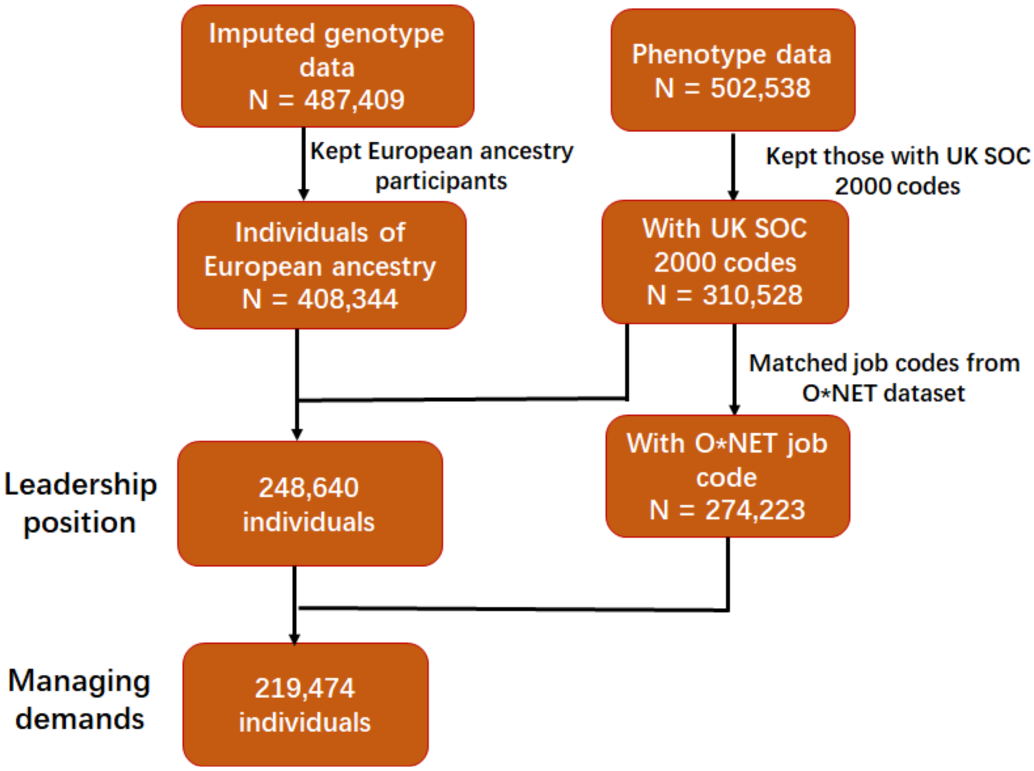

Supplementary Figure 1. Data preparation for the U.K. Biobank discovery sample. The current analysis includes U.K. biobank imputed genotype data for the European population only. A total of 248,640 individuals with leadership position phenotype from UK SOC 2000 codes were included. By linking to the O*NET dataset, 219,474 individuals with managing demand phenotypes were included in GWAS analysis.

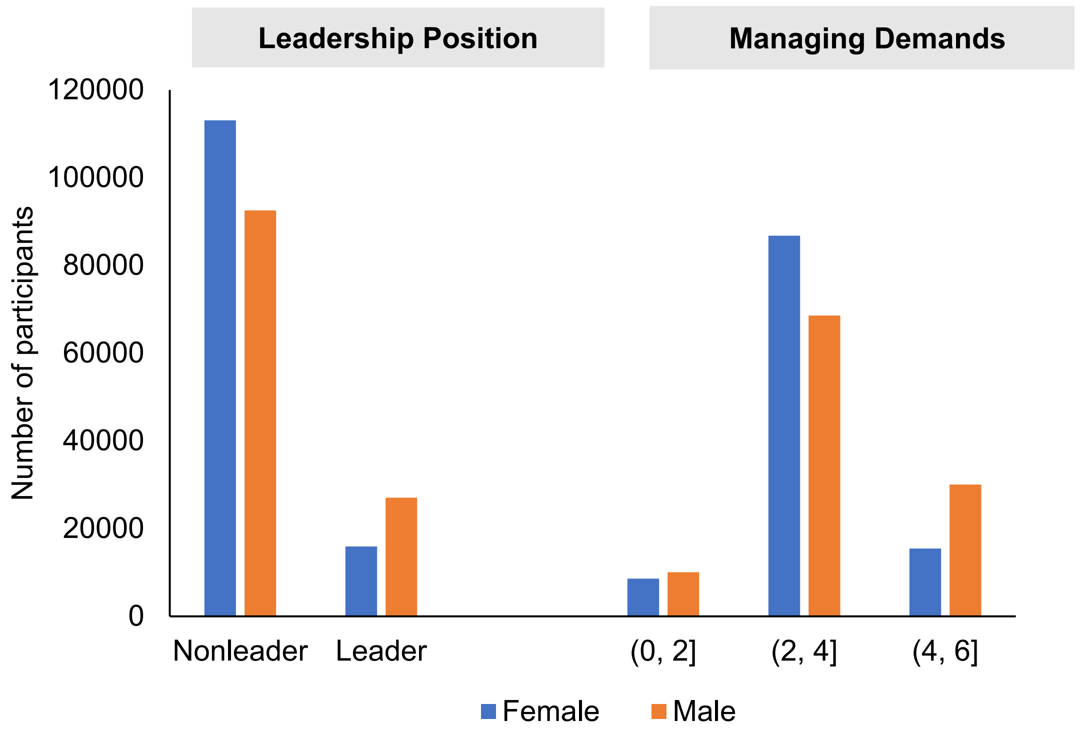

Supplementary Figure 2. Distribution of leadership position and managing demands by sex in the UKB discovery sample.

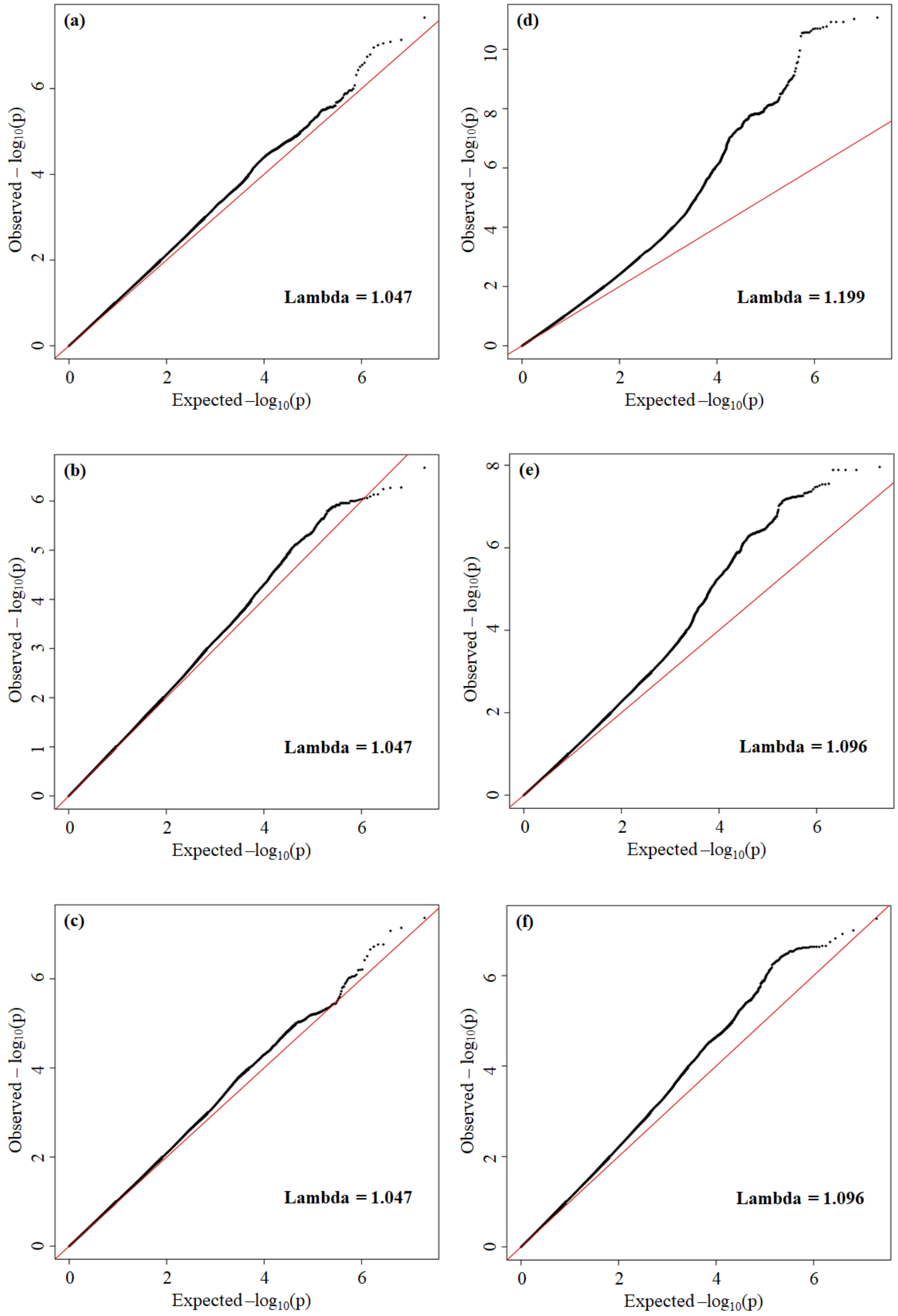

leadership position

Supplementary Figure 3. Quantile-quantile plots with genomic control inflation factor lambda λ_G (a) leadership position; (b) leadership position (female); (c) leadership position (male); (d) managing demands; (e) managing demands (female); (f) managing demands (male).

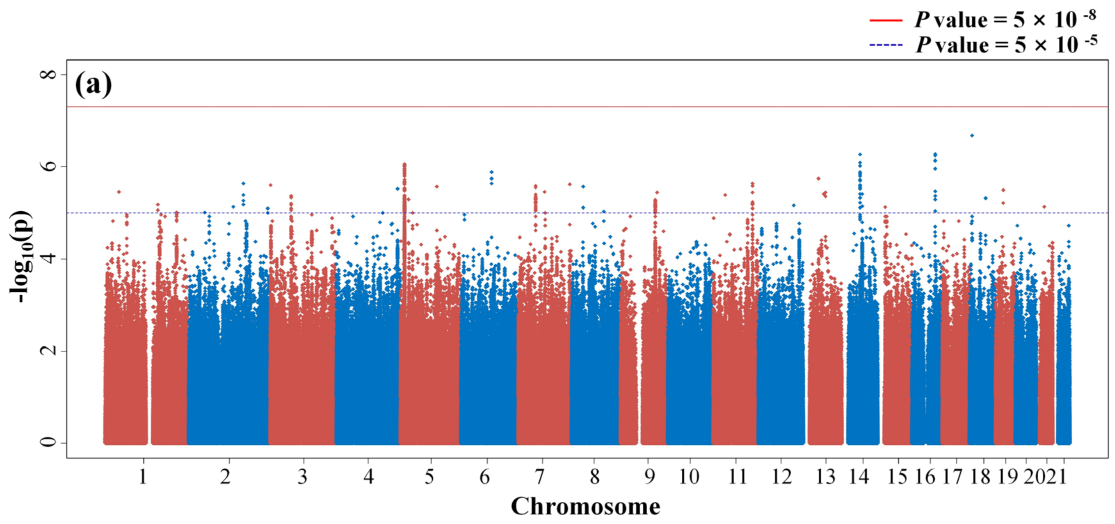
P value = 5 × 10 -8
P value = 5 × 10 -5
(a)
-log10(p)
Chromosome
1 2 3 4 5 6 7 8 9 10 11 12 13 14 15 16 17 18 19 20 21

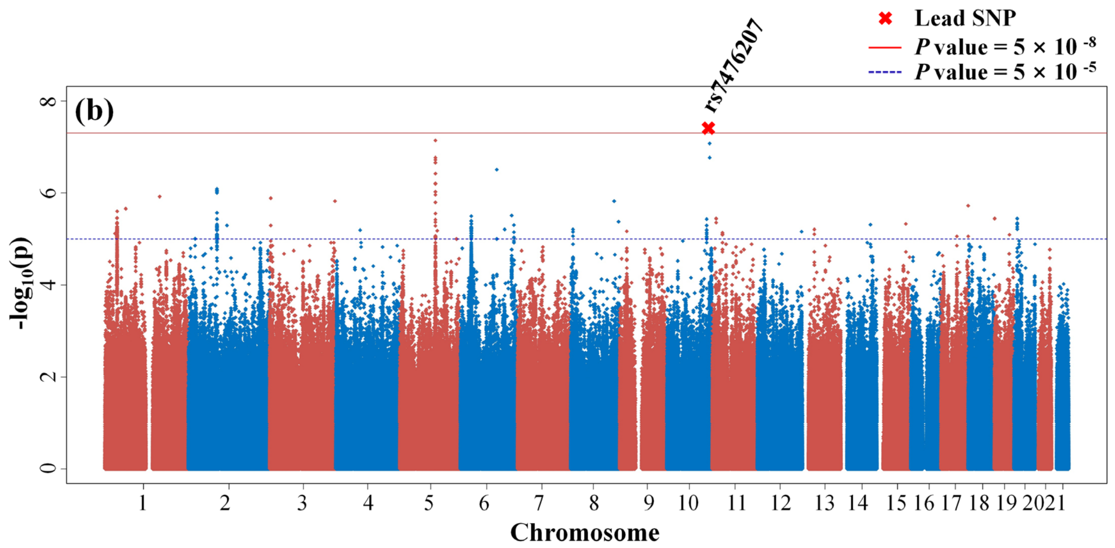
Lead SNP
P value = 5 × 10 -8
P value = 5 × 10 -5
rs7476207
(b)
-log10(p)
Chromosome
1 2 3 4 5 6 7 8 9 10 11 12 13 14 15 16 17 18 19 20 21

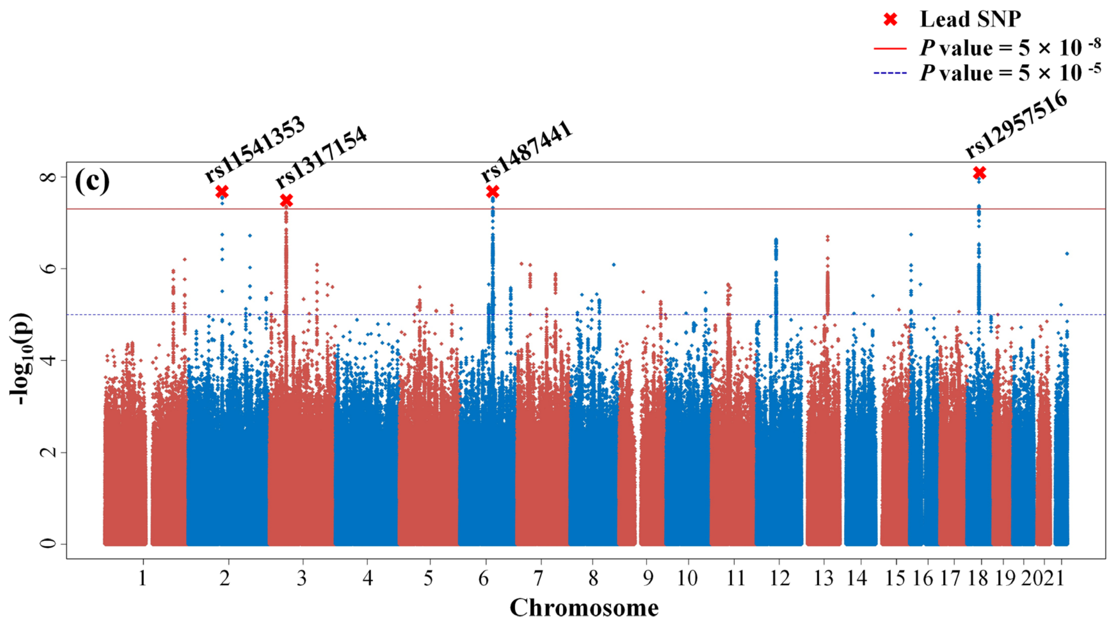

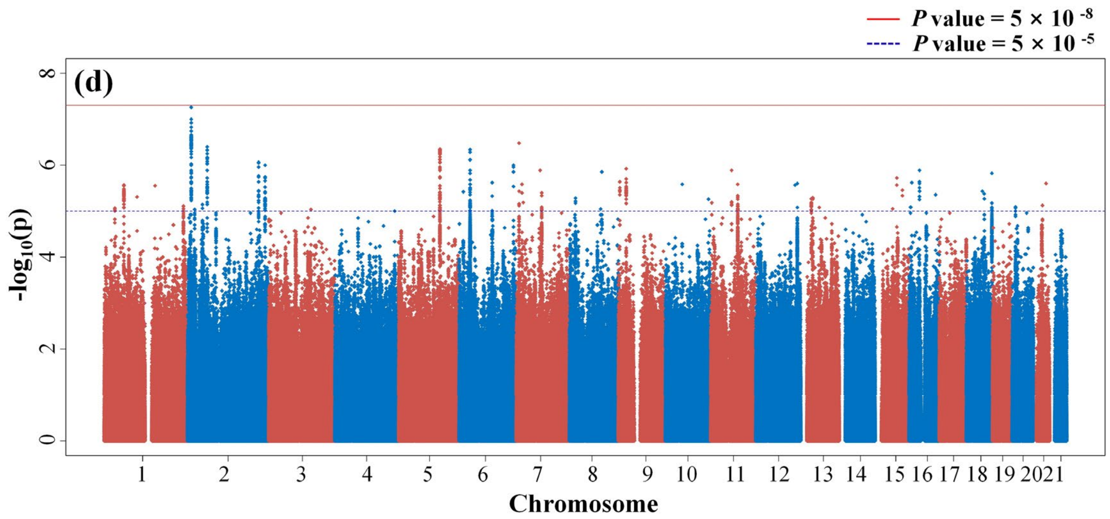

Supplementary Figure 4. Manhattan plot of GWAS analysis for (a) leadership position (female); (b) leadership position (male); (c) managing demands (female); (d) managing demands (male). For c), 4 independent loci were reaching genome-wide significance for managing demands in females. Two loci at chromosome 3 (lead SNP rs1317154) and chromosome 6 (lead SNP rs1487441) are overlapping with the loci identified for the whole sample. rs11541353 at chromosome 1 and rs12957516 at chromosome were two additional loci identified for management demands in females. A total of 3 additional loci were identified in across-sex analyses.

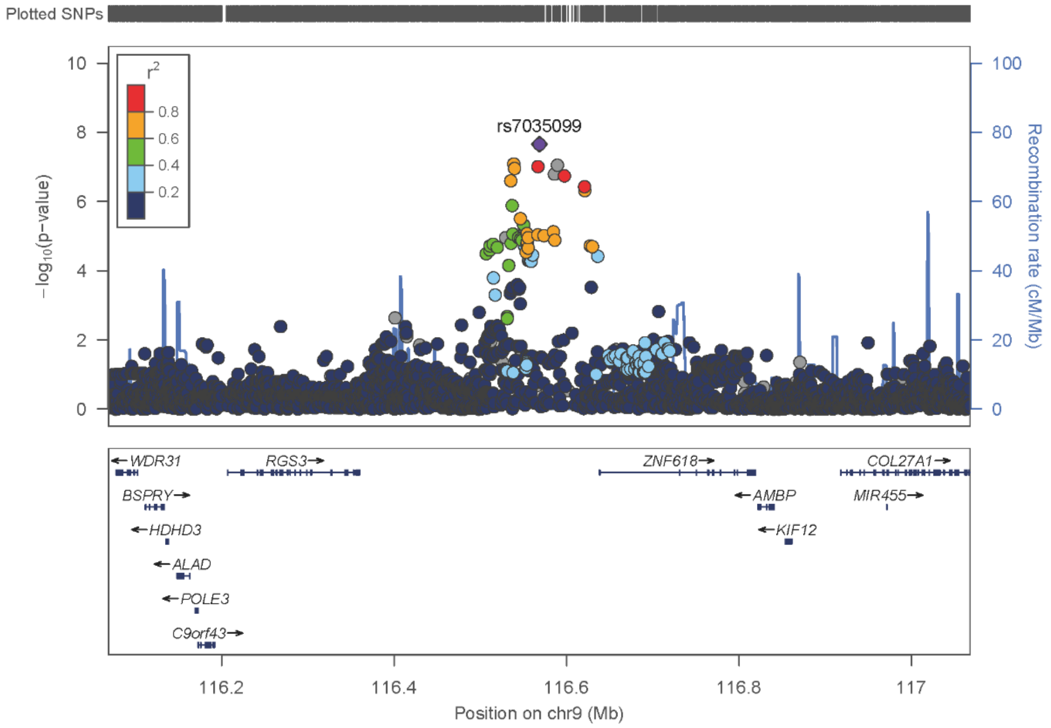
Plotted SNPs
rs7035099
r2
0.8
0.6
0.4
0.2
−log10(p−value)
Recombination rate (cM/Mb)
WDR31
RGS3
ZNF618
COL27A1
BSPRY
AMBP
MIR455
HDHD3
KIF12
ALAD
POLE3
C9orf43
116.2
116.4
116.6
116.8
117
Position on chr9 (Mb)

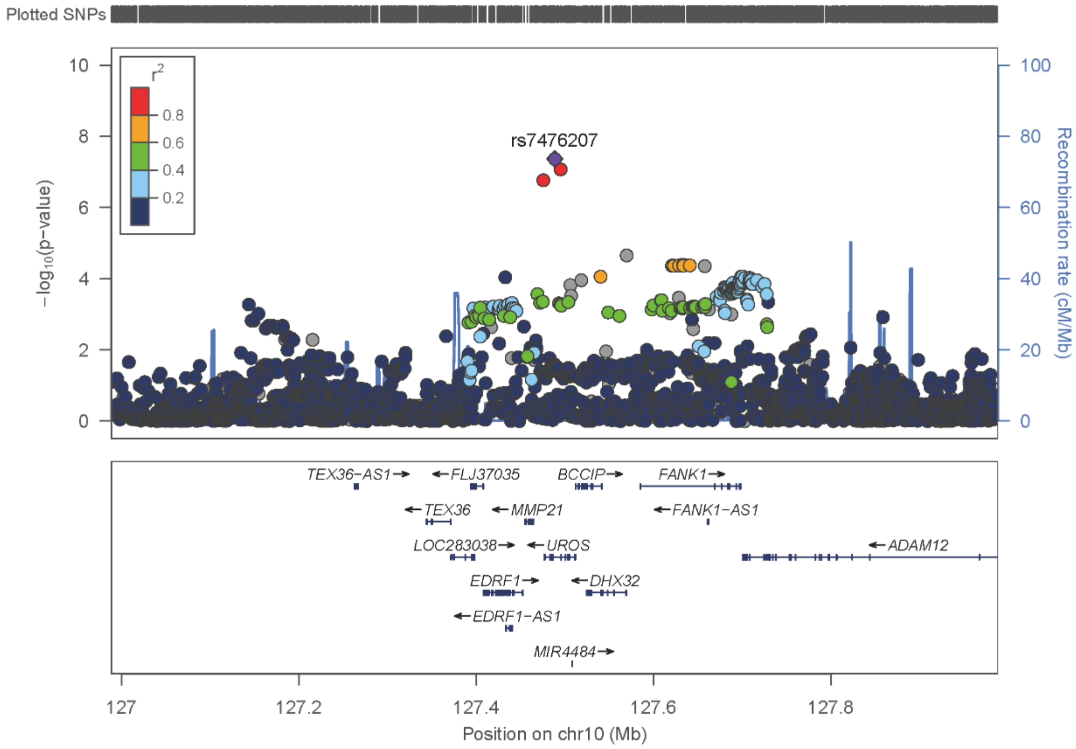
Plotted SNPs
rs7476207
r2
0.8
0.6
0.4
0.2
−log10(p−value)
Recombination rate (cM/Mb)
TEX36−AS1
FLJ37035
BCCIP
FANK1
TEX36
MMP21
FANK1−AS1
LOC283038
UROS
ADAM12
EDRF1
DHX32
EDRF1−AS1
MIR4484
127
127.2
127.4
127.6
127.8
Position on chr10 (Mb)

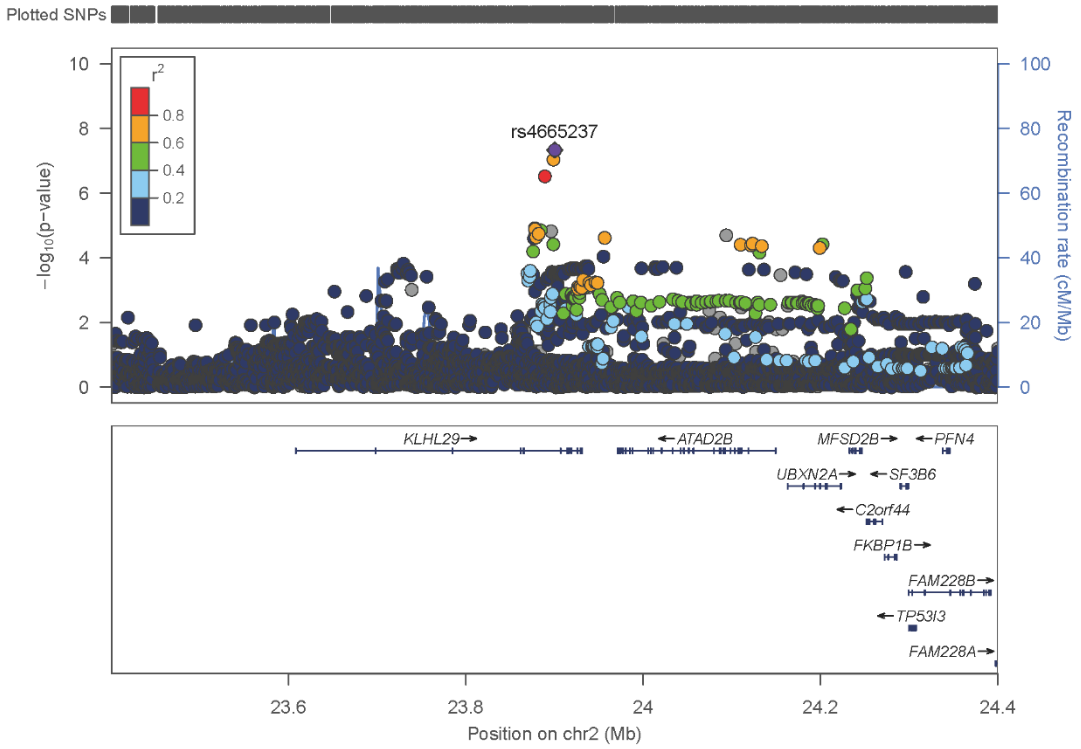
Plotted SNPs
r²
0.8
0.6
0.4
0.2
rs4665237
−log10(p−value)
Recombination rate (cM/Mb)
KLHL29
ATAD2B
MFSD2B
PFN4
UBXN2A
SF3B6
C2orf44
FKBP1B
FAM228B
TP53I3
FAM228A
23.6
23.8
24
24.2
24.4
Position on chr2 (Mb)

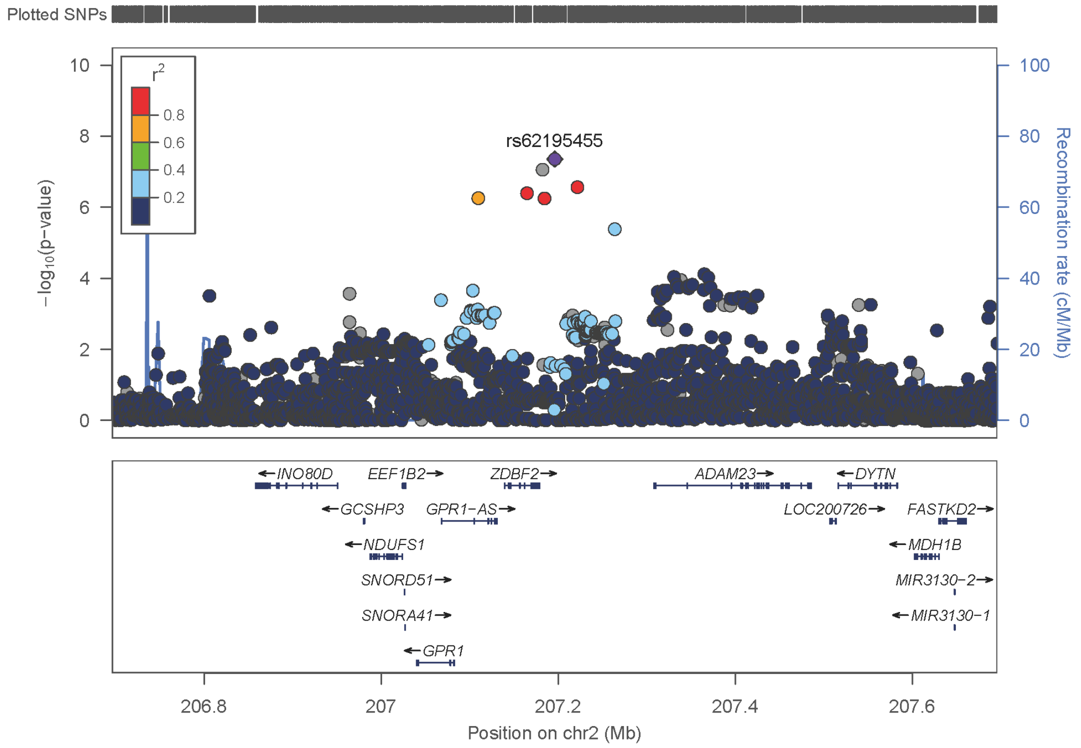
Plotted SNPs
r²
0.8
0.6
0.4
0.2
rs62195455
−log10(p−value)
Recombination rate (cM/Mb)
INO80D
EEF1B2
ZDBF2
ADAM23
DYTN
GCSHP3
GPR1−AS
LOC200726
FASTKD2
NDUFS1
MDH1B
SNORD51
MIR3130−2
SNORA41
MIR3130−1
GPR1
206.8
207
207.2
207.4
207.6
Position on chr2 (Mb)

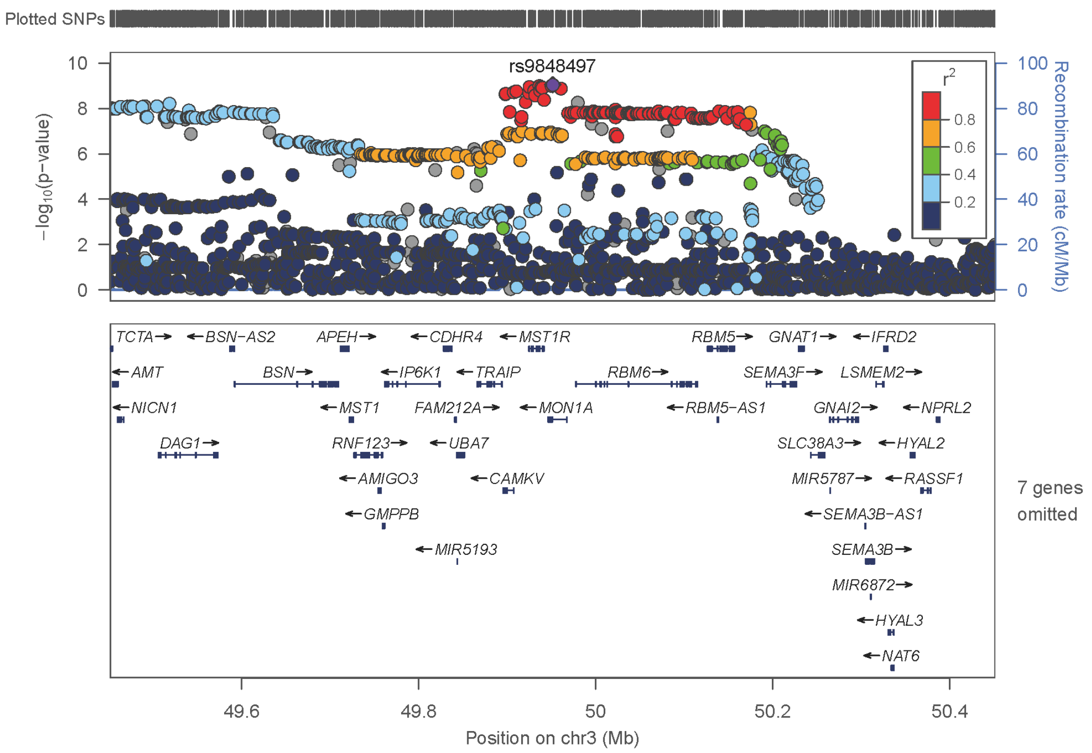
Plotted SNPs
rs9848497
−log10(p−value)
Recombination rate (cM/Mb)
r2
0.8
0.6
0.4
0.2
TCTA
BSN−AS2
APEH
CDHR4
MST1R
RBM5
GNAT1
IFRD2
AMT
BSN
IP6K1
TRAIP
RBM6
SEMA3F
LSMEM2
NICN1
MST1
FAM212A
MON1A
RBM5−AS1
GNAI2
NPRL2
DAG1
RNF123
UBA7
SLC38A3
HYAL2
AMIGO3
CAMKV
MIR5787
RASSF1
GMPPB
SEMA3B−AS1
MIR5193
SEMA3B
MIR6872
HYAL3
NAT6
7 genes omitted
Position on chr3 (Mb)

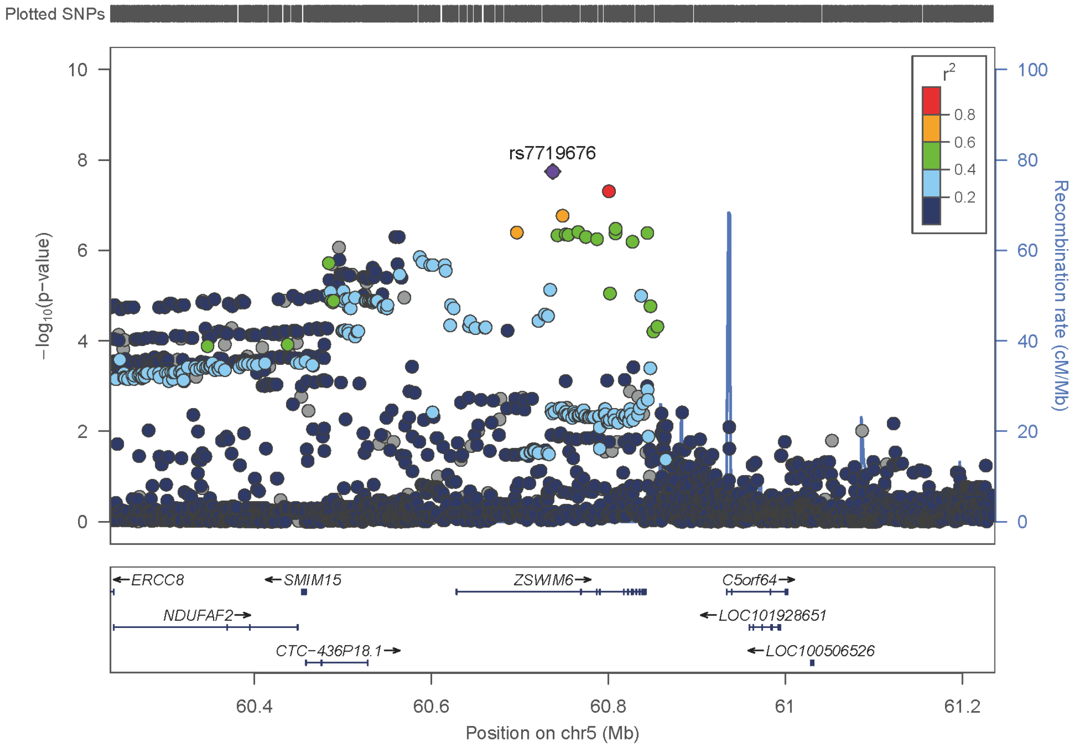
Plotted SNPs
rs7719676
−log10(p−value)
Recombination rate (cM/Mb)
r2
0.8
0.6
0.4
0.2
ERCC8
SMIM15
ZSWIM6
C5orf64
NDUFAF2
LOC101928651
CTC−436P18.1
LOC100506526
Position on chr5 (Mb)

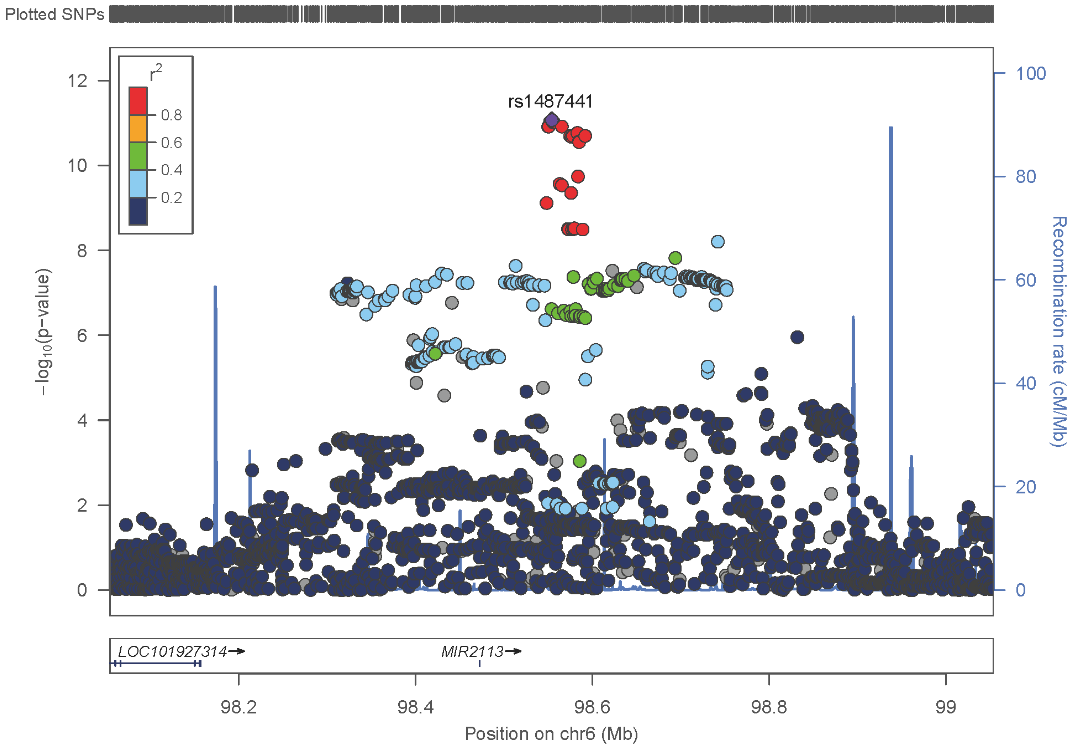
Plotted SNPs
rs1487441
r2
0.8
0.6
0.4
0.2
−log10(p−value)
Recombination rate (cM/Mb)
LOC101927314
MIR2113
98.2
98.4
98.6
98.8
99
Position on chr6 (Mb)

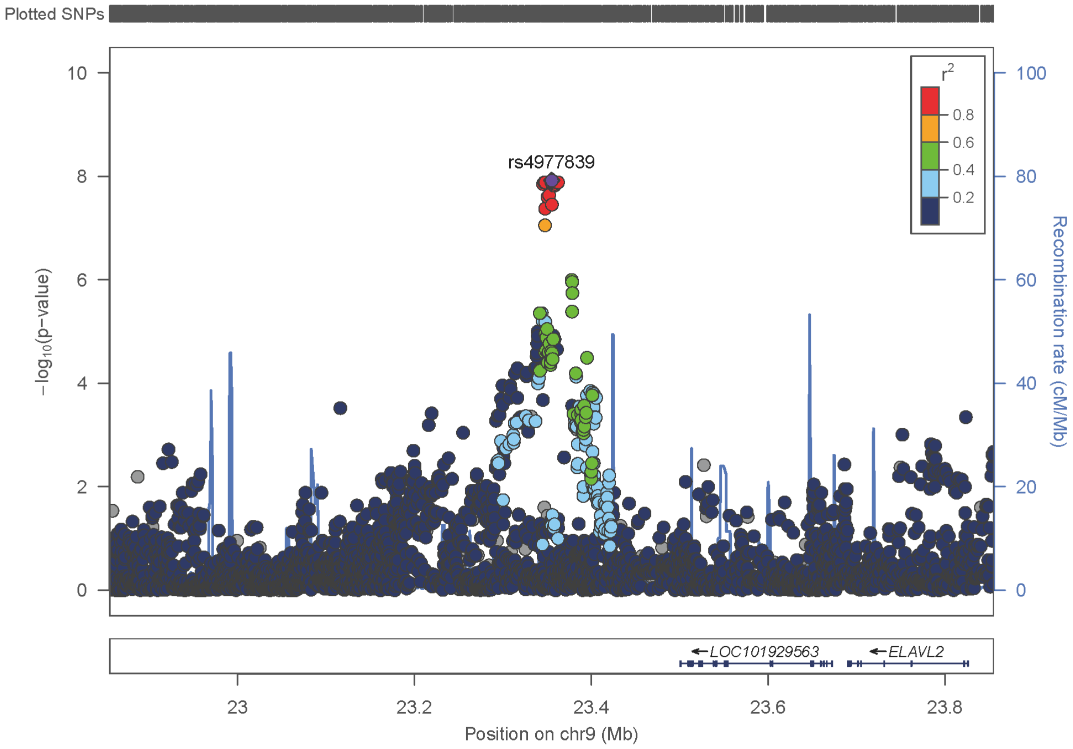
Plotted SNPs
rs4977839
r2
0.8
0.6
0.4
0.2
−log10(p−value)
Recombination rate (cM/Mb)
LOC101929563
ELAVL2
23
23.2
23.4
23.6
23.8
Position on chr9 (Mb)

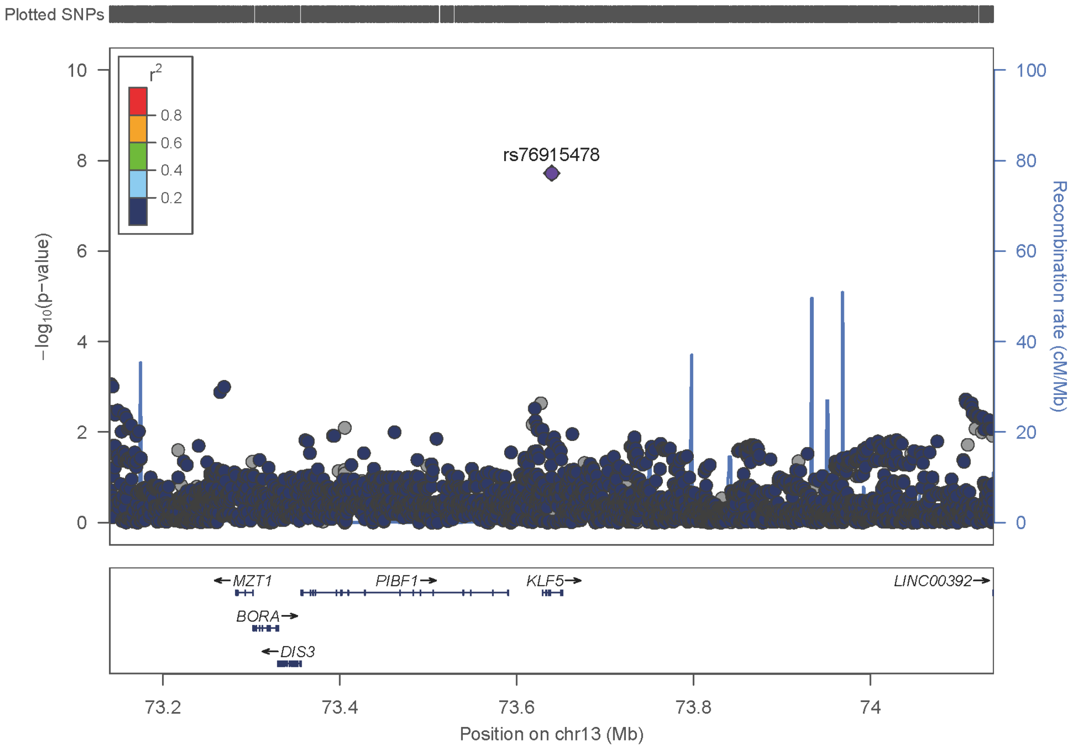
Plotted SNPs
r2
0.8
0.6
0.4
0.2
rs76915478
−log10(p−value)
Recombination rate (cM/Mb)
MZT1
PIBF1
KLF5
LINC00392
BORA
DIS3
73.2
73.4
73.6
73.8
74
Position on chr13 (Mb)

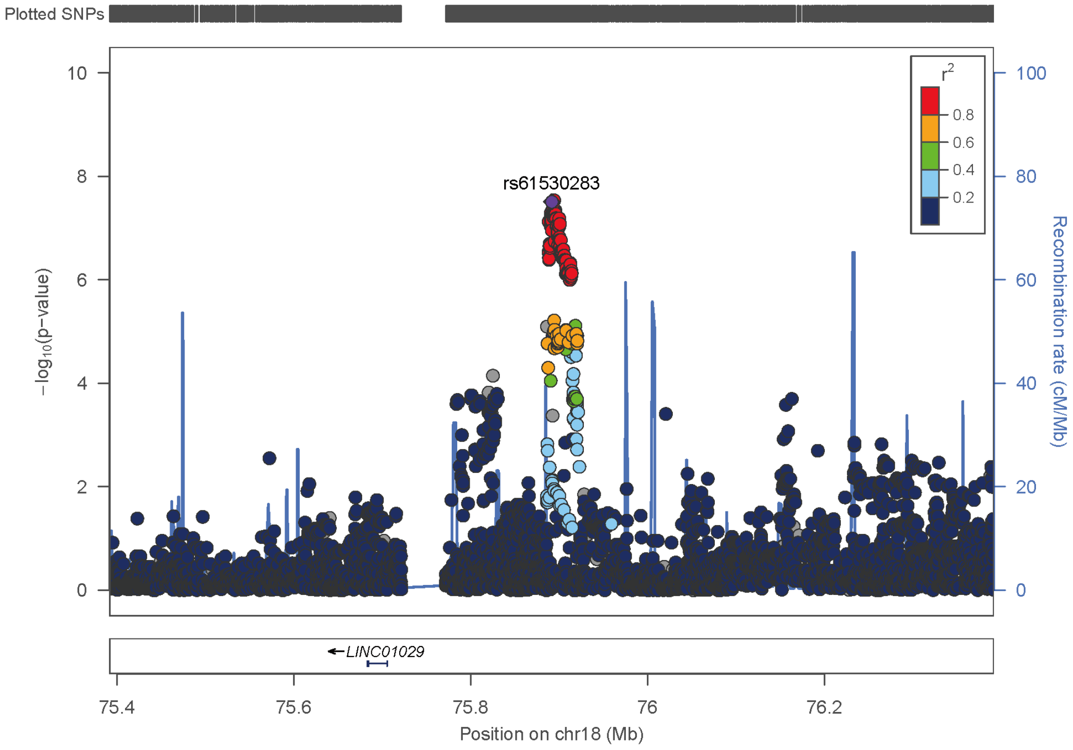
Plotted SNPs
r2
0.8
0.6
0.4
0.2
rs61530283
−log10(p−value)
Recombination rate (cM/Mb)
LINC01029
75.4
75.6
75.8
76
76.2
Position on chr18 (Mb)

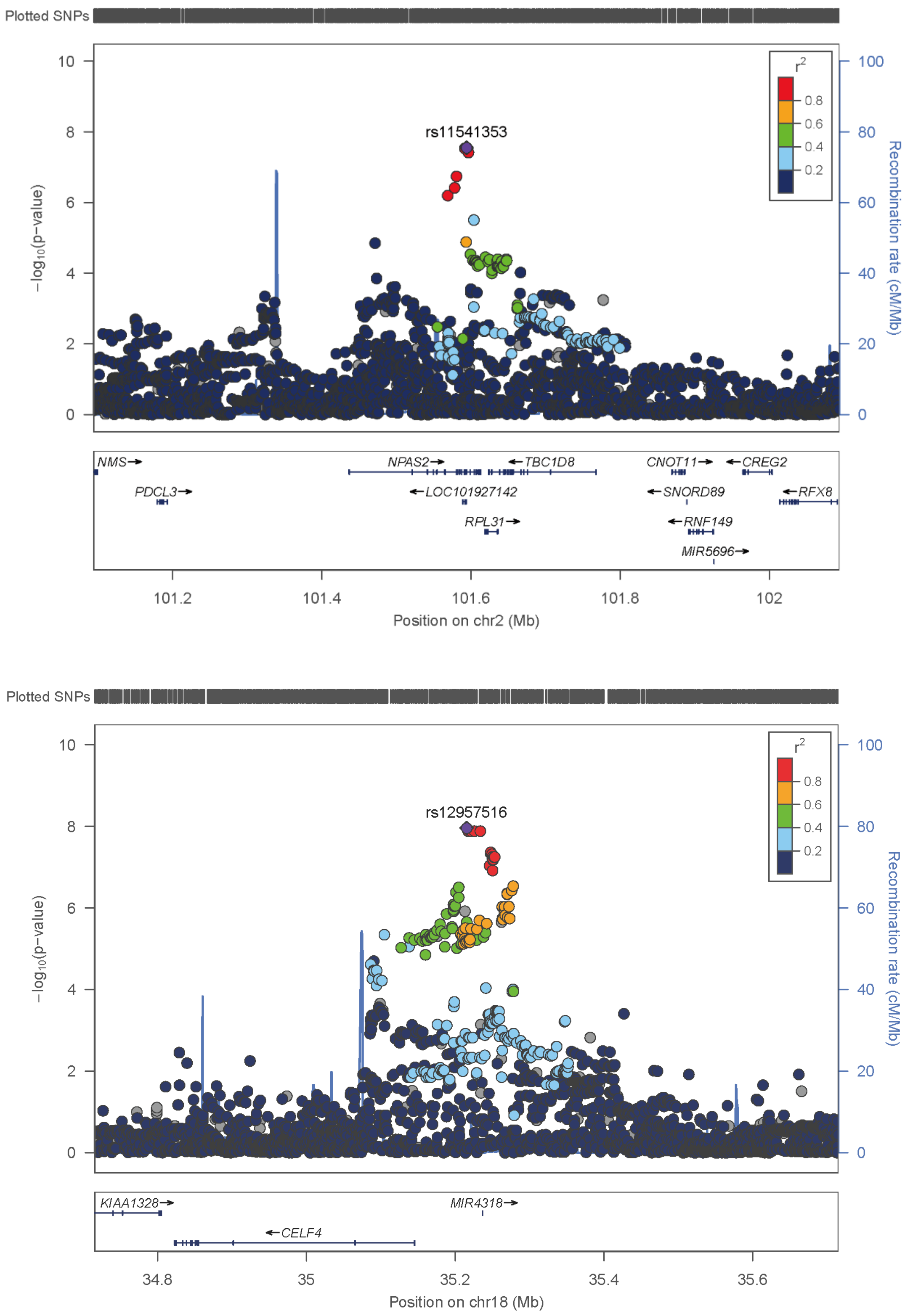

Supplementary Figure 5. Regional plots for leadership position and managing demands

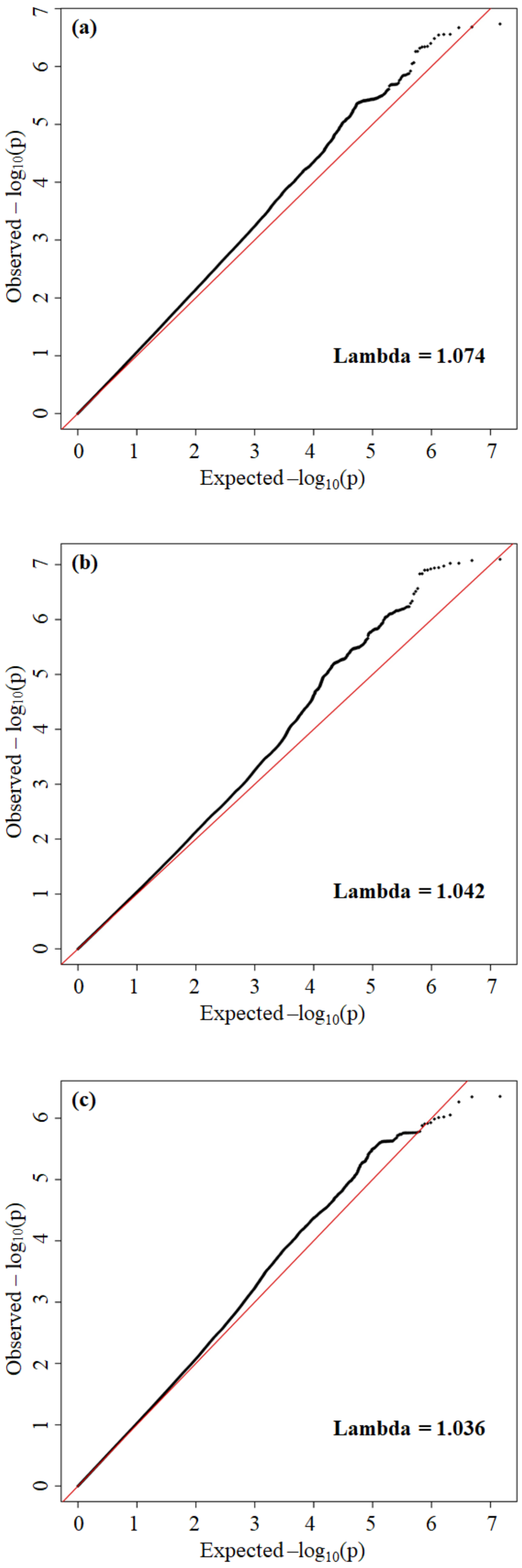

Supplementary Figure 6. Quantile-quantile plots for MTAG-leadership in the UKB discovery sample. (a) all sample; (b) female; (c) male.

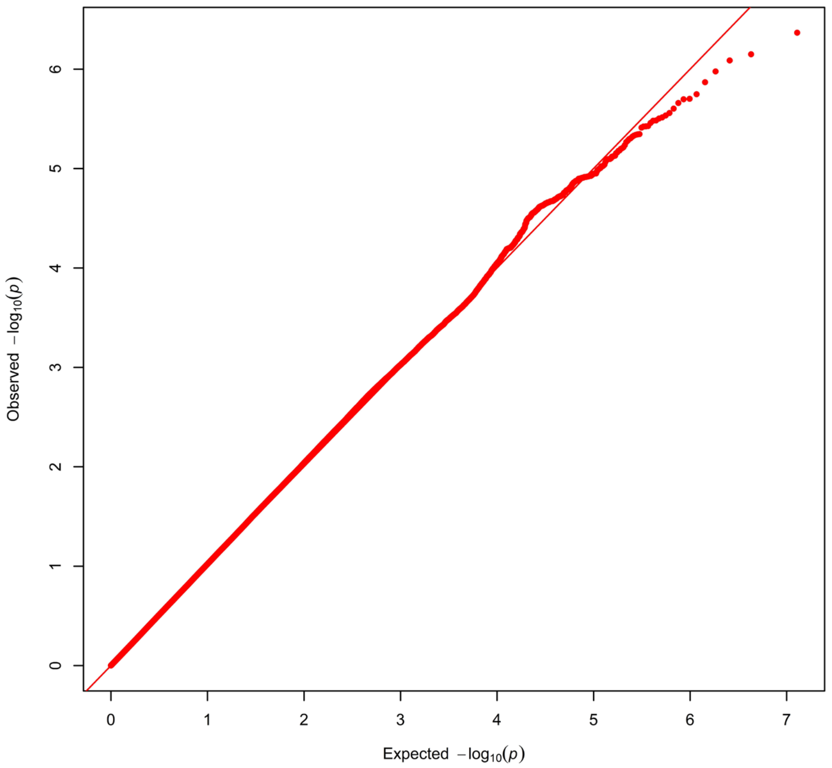

Supplementary Figure 7. Quantile-quantile plot of GWAS meta-analysis results for senior leadership across UKB (discovery and follow-up datasets) and the Add Health samples.
The genomic control lambda = 1.027.

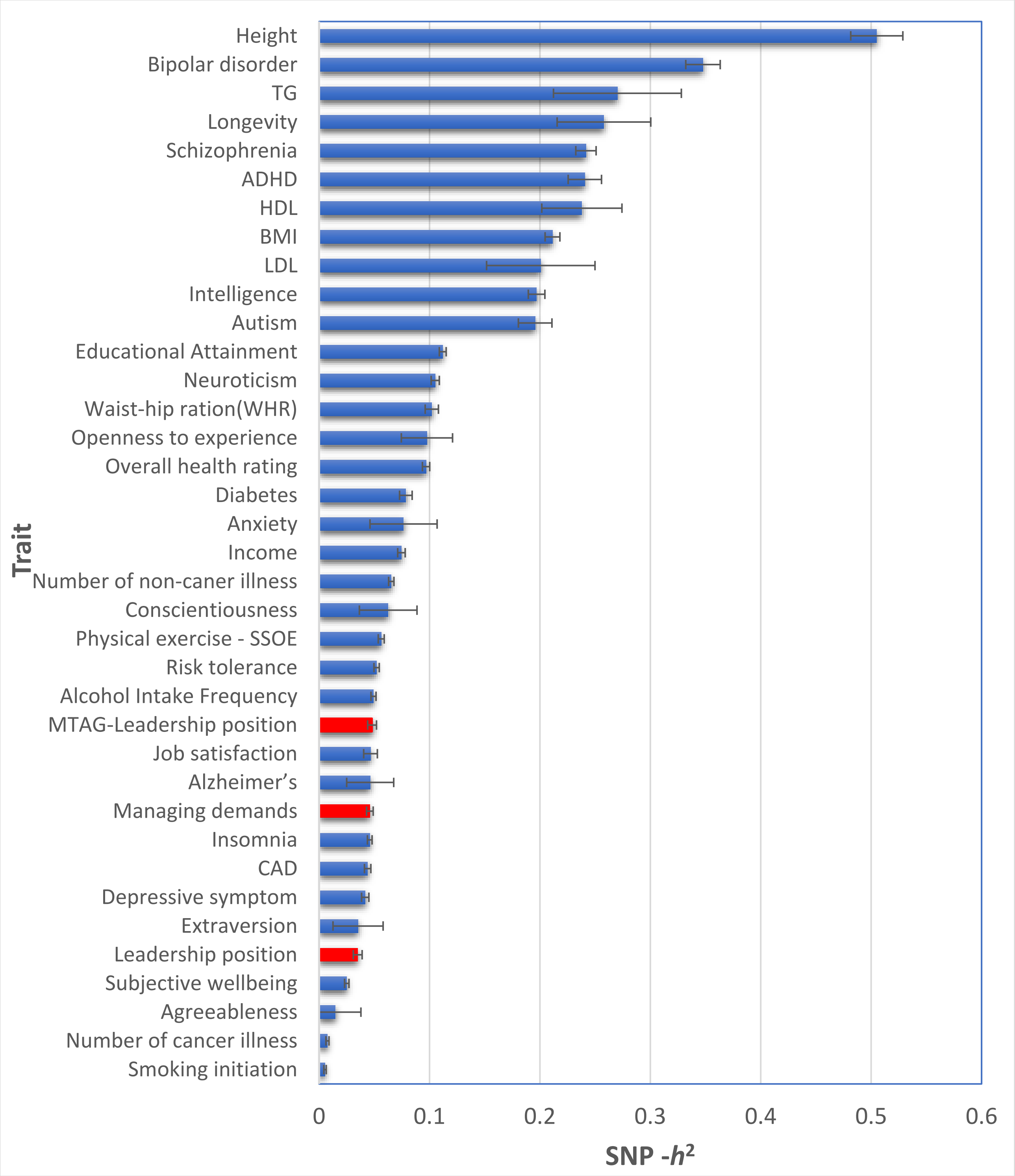

Figure S8. **SNP-h^2 estimates for leadership position phenotypes, compared with a variety of other traits.**

LDSC approach was used for the estimation.

ADHD – Attention Deficit Hyperactivity Disorder, CAD – Coronary Artery Disease, BMI – Body Mass Index, WHR – Whist-hip Ratio, HDL – High-density Lipoprotein, LDL – Low-density lipoprotein, TG – Triglycerides.

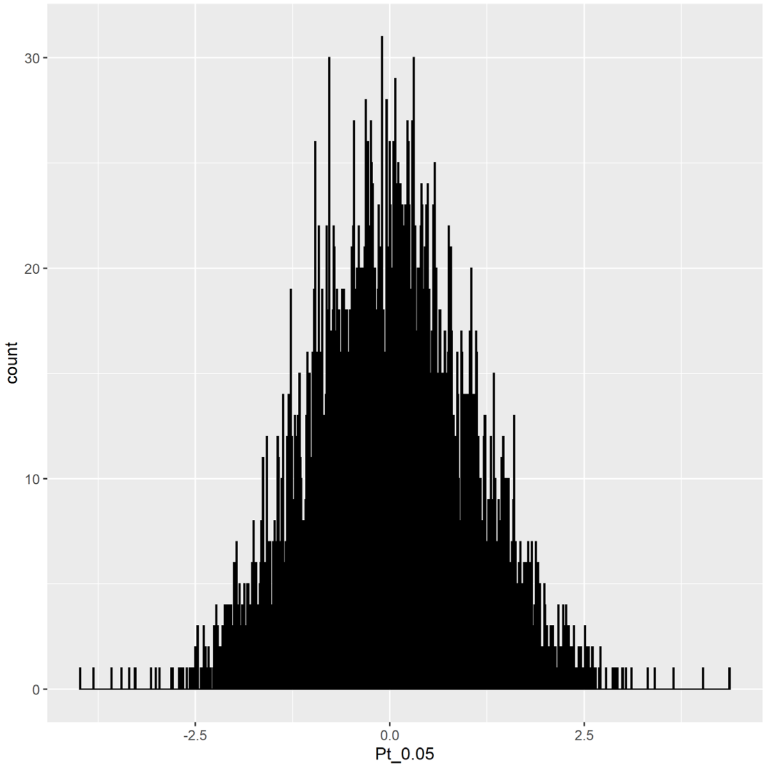

Supplementary Figure 9. Distribution of PGS constructed in the UKB follow-up cohort. The sample size was 22,875; the P-value threshold is set at 0.05.

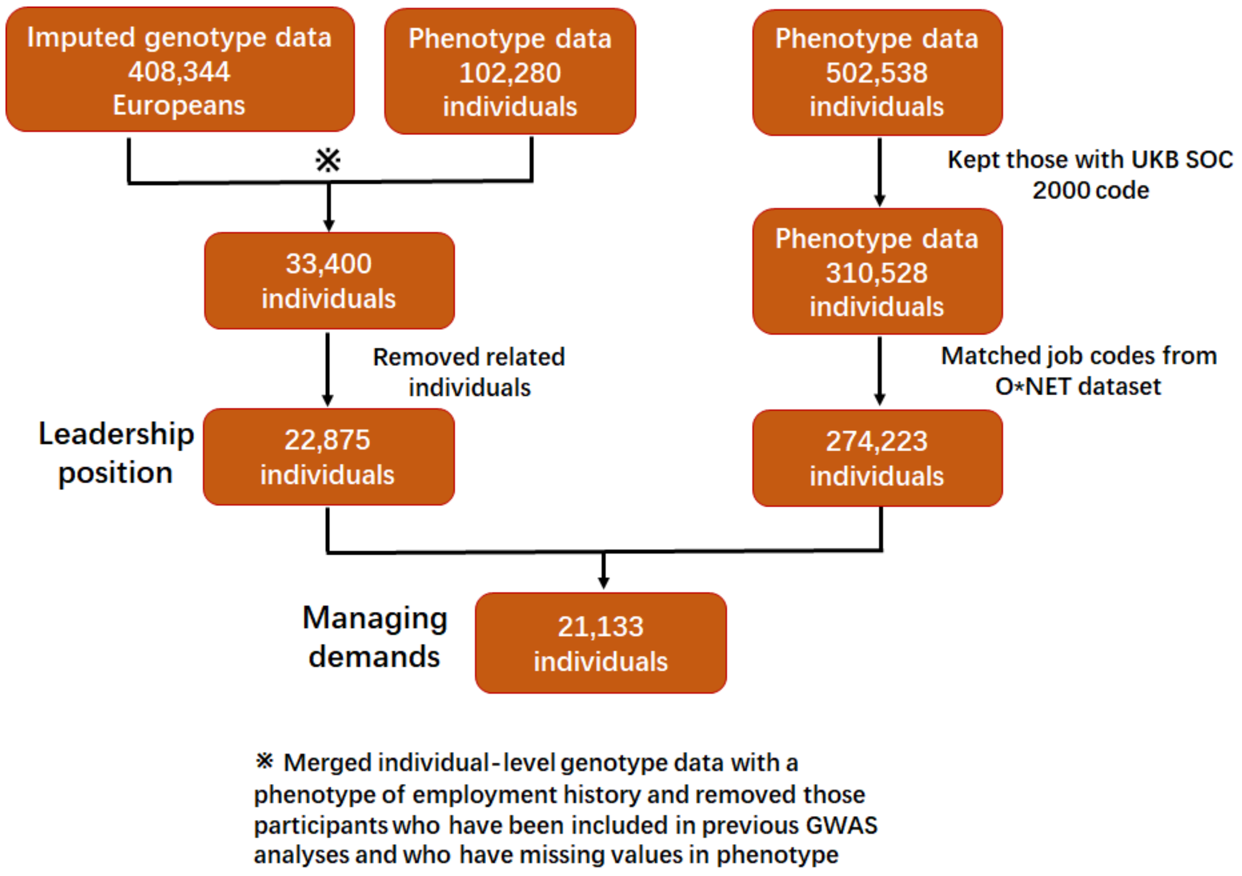

Supplementary Figure 10. Data preparation for the U.K. Biobank follow-up sample. The replication samples comprised 22,875 individuals for leadership position and 21,133 individuals for managing demands with genome data and valid job information of the last job, but not included in the discovery phase.

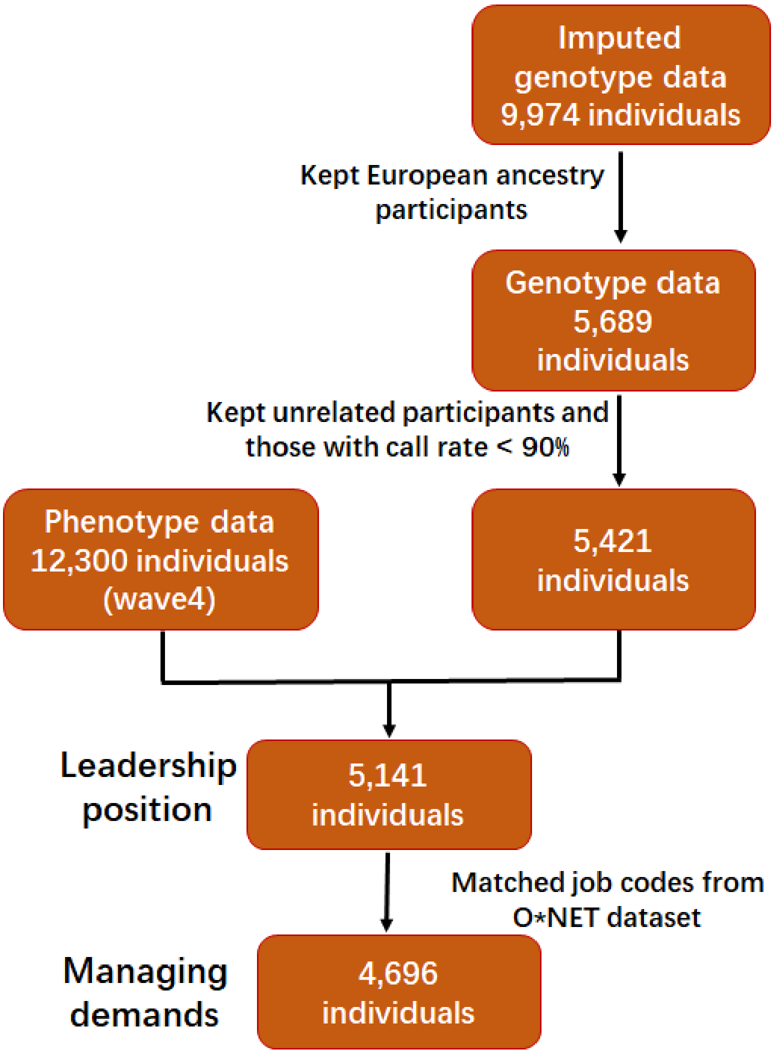

Supplementary Figure 11. Data preparation for the Add Health Wave IV data.
The replication stage includes unrelated individuals of European ancestry from the Add Health Wave 4 data, with imputed markers, referenced to the Haplotype Reference Consortium panel (HRCr1.1). 5,141 individuals were available for leadership position and 4,696 individuals were available for managing demands.

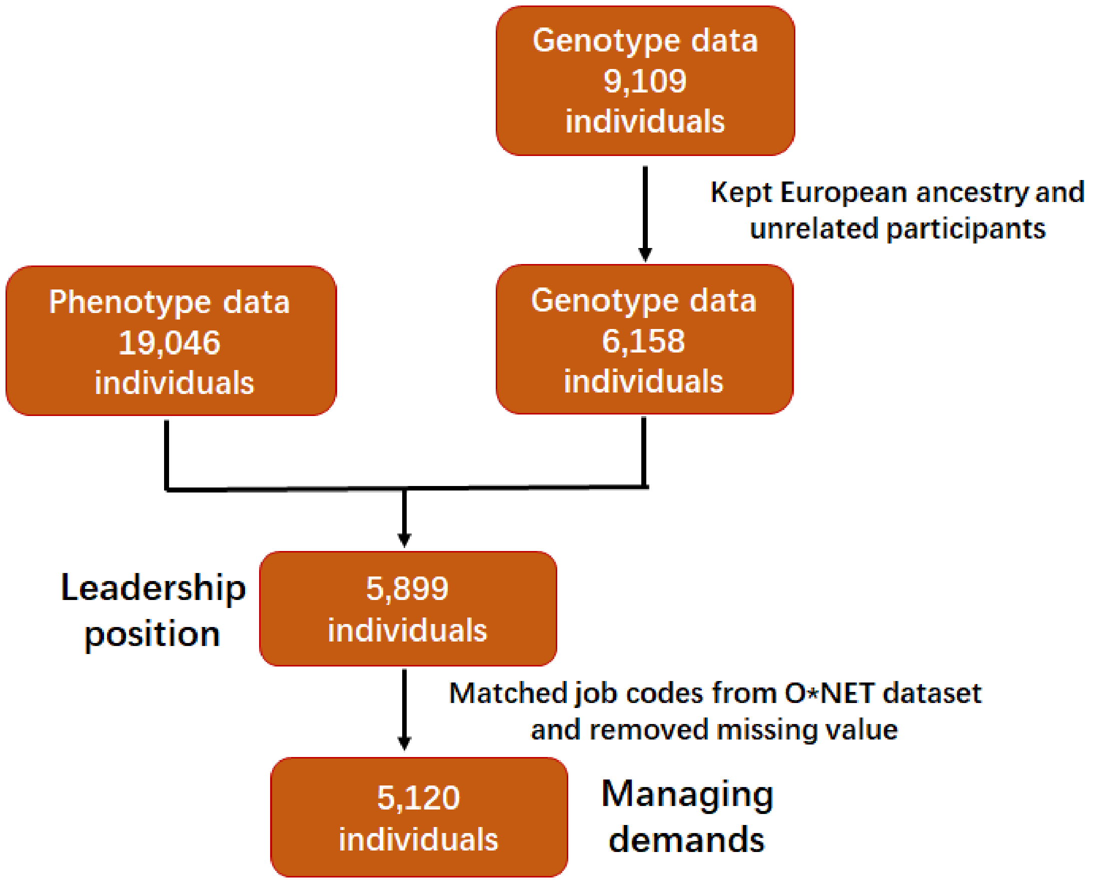

Supplementary Figure 12. Data preparation for the Wisconsin Longitudinal Study data. The replication stage included unrelated individuals of European ancestry from the Wisconsin Longitudinal Study data. 5,899 individuals were available for leadership position and 5,120 individuals were available for managing demands.

Supplementary Table 1. Phenotypic description of leadership position and detailed items of managing demands

Variable name	Source	Description	How to code
Leadership position	UK SOC 2000 Job codes	This indicator was derived from occupational codes with the UK SOC 2000 system. Occupations that required managing subordinates were coded as holding leadership positions (1) and those with other occupations were coded as non-leaders (0). Such sample leadership occupations include “directors and chief executives of major organizations” and “Marketing and sales managers”. Sample non-leader job titles were “Sales representatives” and “artists”.	0, 1
Managing demands	(*7*)	1. Scheduling Work and Activities (Scheduling events, programs, and activities, as well as the work of others).	7-point scale: 1 = lowest level, 7= highest level
		2. Resolving Conflicts and Negotiating with Others (Handling complaints, settling disputes, and resolving grievances and conflicts, or otherwise negotiating with others)	
		3. Coordinating the Work and Activities of Others (Getting members of a group to work together to accomplish tasks.)	
		4. Developing and Building Teams (Encouraging and building mutual trust, respect, and cooperation among team members.)	
		5. Guiding, Directing, and Motivating Subordinates (Providing guidance and direction to subordinates, including setting performance standards and monitoring performance.)	
		6. Coaching and Developing Others (Identifying the developmental needs of others and coaching, mentoring, or otherwise helping others to improve their knowledge or skills.)	
		7. Performing Administrative Activities (Performing day-to-day administrative tasks such as maintaining information files and processing paperwork.)	
		8. Staffing Organizational Units (Recruiting, interviewing, selecting, hiring, and promoting employees in an organization.)	
		9. Monitoring and Controlling Resources (Monitoring and controlling resources and overseeing the spending of money.)	

Note. The measure of managing demands was presented as the average score across these 9 items

Supplementary Table 2. Summary of O*NET linked phenotype scores of managing demands in the U.K. Biobank discovery data

	N	Mean (SD)
Age	248,640	54.33 (7.66)
Managing demands	219,474	3.13 (0.92)
Male leaders	27,066	4.43 (0.43)
Male non-leaders	92,552	2.92 (0.77)
Female leaders	15,932	4.38 (0.52)
Female non-leaders	113,090	2.82 (0.75)

Note. Mean (S.D.) and Range for Male/Female (non-) leaders were mean (S.D.) of managing demands by their leadership positions.

Supplementary Table 3. Phenotypic correlation between leadership position and managing demands

	Trait1	Trait2	Phenotypic correlation
All	Leadership position	Managing demands	.626**
Female	Leadership position	Managing demands	.572**
Male	Leadership position	Managing demands	.659**

Supplementary Table 4. Summary of personal information in the U.K. Biobank discovery data

Variables	N	Leaders (%) (N= 42,998)	Non-leaders (%) N=205,642	*P*
Education				
College or University degree	86555	37.94	34.16	<0.001
A/AS, NVQ or HND or HNC or equivalent, Other professional qualifications	58489	25.54	23.10	
O levels/GCSEs, CSEs or equivalent	72311	28.74	29.15	
None of the above, prefer not to answer	31062	7.70	13.49	
Missing	223	0.08	0.09	
Average total household income before tax				
Greater than 100,000	14896	11.93	4.75	<0.001
52,000 to 100,000	59310	35.95	21.32	
31,000 to 51,999	68502	27.40	27.58	
18,000 to 30,999	52244	12.94	22.70	
Less than 18,000	28261	4.61	12.78	
Missing	25427	7.16	10.87	
Townsend deprivation index at recruitment, SD	-1.60 (2.83)	-1.98 (2.65)	-1.51 (2.86)	<0.001
Own or rent accommodation lived in				
Own	229037	94.64	91.59	<0.001
Rent	17122	4.62	7.36	
Missing	2481	0.74	1.05	
Number of vehicles in household				
None	13454	2.75	5.97	<0.001
One	92729	30.58	38.70	
Two and above	141632	66.43	54.98	
Missing	825	0.25	0.35	
Transport type for commuting to workplace				
Car/motor vehicle	163481	70.09	64.84	<0.001
Walk	17856	5.05	7.63	
Public transport	16385	5.52	6.81	
Cycle	4450	1.21	1.91	
Missing	46468	18.13	18.81	

Chi-square test was performed to compare the proportions between leaders vs. non-leader
Two-sample t-test was performed for the continuous variables

Supplementary Table 5. Sample size and demographic distribution in replication samples

	UKB follow-up dataset	Add Health dataset	Wisconsin Longitudinal Study (WLS)
**leadership position**	N=22,875	N=5,141	N=5,899
**Leaders (%)**	3,639 (15.90)	1,956 (38.04)	
**Non-leaders (%)**	19,236 (84.10)	3,185 (61.96)	
**Leadership position score**			
**mean (sd)**			0.37 (0.42)
**Male %**	10,028 (43.84)	2,406 (46.80)	2,903 (49.21)
**Age**			
**years (sd)**	60.39 (6.42)	28.38 (1.78)	68.62(2.40)
**Managing demands**	N=21,133	N=4,696	N=5,120
**mean (sd)**	3.27(0.87)	3.01(0.80)	2.99(0.75)

For UKB and Add Health datasets, the leadership position is a dichotomous variable; For WLS, the leadership position variable is a score representing the level of leadership position. sd- standard deviation.

Supplementary Table 6. Genomic control λ for GWAS on leadership traits in UK Biobank data

	λ	LDSC Intercept (s.e.)
leadership position	1.047	1.0033 (0.0066)
leadership position(female)	1.047	1.0054 (0.0067)
leadership position(male)	1.047	1.0105 (0.0062)
managing demands	1.199	1.0534 (0.0078)
managing demands(female)	1.096	1.017 (0.0072)
managing demands(male)	1.096	1.0459 (0.0072)

LDSC intercept - Intercepts of univariate linkage disequilibrium score regression (LDSC)

Supplementary Table 7. GWAS top variants for leadership position and managing demands phenotypes and replication

			Replication stage				Meta-analysis of all samples				
SNP	A1/A2	Genes	BETA	s.e	*P*	*HetP*	BETA	s.e	*P*	Direction	*HetP*
1. All sample analyses											
Leadership position (N =33,915)							N = 282,555				
**rs7035099**	T/C	*ZNF618; RGS3*	-0.014	0.007	5.99E-02	0.378	-0.006	0.001	6.14E-09	--+-	0.391
Managing demands (N=30,949)							N= 250,423				
**rs4665237**	T/G	*KLHL29*	0.014	0.007	3.89E-02	0.480	0.015	0.003	5.22E-09	++-+	0.683
rs62195455	A/G	*ZDBF2; ADAM23*	0.001	0.010	9.30E-01	0.851	-0.019	0.004	4.93E-07	--+-	0.172
**rs9848497**	T/C	*MST1R;MON1A*	0.006	0.007	4.07E-01	0.617	0.015	0.003	2.21E-09	++++	0.330
**rs7719676**	A/G	*ZSWIM6*	0.011	0.007	1.14E-01	0.816	0.016	0.003	6.53E-09	++++	0.827
**rs1487441**	A/G	*MIR2113; POU3F2*	0.016	0.007	1.75E-02	0.824	0.018	0.003	5.17E-13	++++	0.908
**rs4977839**	A/G	*LINC01239:ELAVL2*	0.008	0.007	2.55E-01	0.852	0.015	0.003	1.20E-08	++++	0.664
**rs76915478**	A/G	*KLF5*	-0.030	0.013	1.86E-02	0.161	-0.029	0.005	1.10E-09	---+	0.301
**rs61530283**	A/G	*LINC01029*	0.008	0.008	2.74E-01	0.396	0.016	0.003	3.09E-08	+++-	0.383
2. Sex-specific analyses											
Leadership position -Male											
rs7476207	C/G	*UROS*	-0.014	0.017	4.23E-01	0.885	0.014	0.003	1.24E-07	+---	0.404
Managing demands - Female											
**rs11541353**	T/C	*NPAS2*	-0.012	0.011	2.80E-01	0.343	-0.025	0.005	3.15E-08	---+	0.312
rs12957516	T/C	*MIR4318; CELF4*	-0.002	0.010	8.50E-01	0.668	0.020	0.004	2.24E-07	+-+-	0.085

A1, effect allele; A2, reference allele; BETA, beta effect based on the effect allele A1. *HetP*, heterogeneity *p*-value.
9 SNPs highlighted in bold with meta-analysis *p*-value across all samples exceeding genome-wide significance were presented in Table 2.
Replication samples included three studies: UKB follow-up cohort, Add Health study, and Wisconsin Longitudinal study.
For the meta-analysis, the sign of direction is in the order of UKB discovery, UKB follow-up cohort, Add Health study, and Wisconsin Longitudinal study.

Supplementary Table 8. Top variants identified for managing demands and cross-trait association analysis on leadership position.

			**Management demands**			**leadership position**			
			N= 219,474			N=282,555			
SNP	Genes	A1/A2	BETA	s.e.	*P*	BETA	s.e.	*P-pleio*	*HetP*
rs4665237	*KLHL29*	T/G	0.015	0.003	4.60E-08	0.002	0.001	6.56E-02	0.634
rs9848497	*MST1R; MON1A*	T/C	0.017	0.003	8.90E-10	0.002	0.001	5.78E-02	0.858
rs7719676	*ZSWIM6*	A/G	0.017	0.003	1.80E-08	0.002	0.001	1.36E-01	0.101
rs1487441	*MIR2113; POU3F2*	A/G	0.019	0.003	8.50E-12	0.004	0.001	3.76E-04	0.283
rs4977839	*LINC01239: ELAVL2*	A/G	0.016	0.003	1.20E-08	0.002	0.001	1.66E-01	0.587
rs76915478	*KLF5*	A/G	-0.029	0.005	1.90E-08	-0.005	0.002	7.77E-03	0.106
rs61530283	*LINC01029*	A/G	0.017	0.003	3.10E-08	0.003	0.001	5.21E-03	0.087
rs11541353	*NPAS2*	T/C	-0.027	0.005	2.80E-08	-0.005	0.002	6.10E-03	0.278

For the variants identified for management demands, the pleiotropic effect refers to the genetic effect for leadership across all datasets. The threshold of significant pleiotropic effect is set to P-pleio at 0.05/8 = 0.00625.

A1, effect allele; A2, reference allele; BETA, beta effect based on the effect allele A1. *HetP*, heterogeneity *p*-value across discovery and replication samples.

Supplementary Table 9. Summary of common SNP heritability estimations for leadership phenotypes from GWAS results in UKB data

**LDSC method**			95% CI					95% CI		
Trait	*Observed - h^2*	S.E.	Lower	Upper	*P*	*Liability-scale h^2*	S.E.	Lower	Upper	*P*
**All**										
Leadership position	0.016	0.002	0.012	0.019	1.67E-18	0.035	0.004	0.027	0.042	5.15E-18
Managing demands	0.046	0.003	0.040	0.052	3.47E-49					
MTAG-leadership	0.022	0.002	0.018	0.026	1.15E-27	0.048	0.004	0.039	0.056	1.36E-28
Senior leadership	0.006	0.003	0.000	0.011	4.55E-02	0.057	0.029	0.001	0.114	4.55E-02
Senior leadership - all datasets	0.007	0.003	0.001	0.013	2.60E-02	0.068	0.030	0.009	0.126	2.49E-02
**Female**										
Leadership position	0.013	0.003	0.006	0.019	1.87E-04	0.033	0.009	0.016	0.050	1.69E-04
Managing demands	0.061	0.005	0.051	0.071	1.75E-31					
MTAG-leadership	0.035	0.004	0.027	0.042	9.40E-22	0.090	0.010	0.071	0.108	3.30E-21
**Male**										
Leadership position	0.015	0.004	0.008	0.022	2.14E-05	0.030	0.007	0.016	0.043	1.79E-05
Managing demands	0.036	0.005	0.026	0.045	1.20E-13					
MTAG-leadership	0.020	0.004	0.013	0.027	6.63E-09	0.039	0.007	0.026	0.053	8.18E-09
**Bolt-REML methods**										
**All**										
Leadership position	0.025	0.003	0.019	0.031	9.67E-16	0.055	0.007	0.041	0.068	9.66E-16
Managing demands	0.078	0.003	0.072	0.085	9.52E-117					
Senior leadership	0.005	0.004	-0.003	0.014	2.37E-01	0.051	0.043	-0.033	0.135	2.37E-01
**Female**										
Leadership position	0.021	0.005	0.011	0.032	4.17E-05	0.055	0.014	0.029	0.082	4.17E-05
Managing demands	0.096	0.006	0.084	0.107	2.21E-63					
**Male**										
Leadership position	0.039	0.005	0.028	0.049	1.80E-12	0.075	0.011	0.054	0.095	1.80E-12
Managing demands	0.095	0.006	0.083	0.106	4.65E-59					

For binary traits, results are reported both on the observed scale and the liability scale. Liability scale SNP-h^2 estimation is preferred for a general population. We estimated the heritability at liability scale (h^2-liability), which equals observed- $h^2 k(1-k)/z^2$ where k is the population prevalence and the denominator is the squared height of the density of the standard normal distribution (mean=0, variance=1) at quantile k (*8*). We regard the population prevalence as the same as the percentage of cases in the dataset. For leadership position phenotype: k=17.29% for all samples, k=22.63% for males and k=12.35% for females. For senior leadership: k=1.593% for UKB; k=2.30% for all datasets (UKB discovery + follow-up +Add Health)
GREML method -- SNP heritability was estimated from GWAS genotype data using software BOLT-LMM v2.3.2

Supplementary Table 10. Genetic correlation for leadership phenotypes across sexes in the UKB sample

	**Male**	**Female**	**Genetic Correlation**	**S.E.**	***P***
	Leadership position	Leadership position	1.08	0.231	3.03E-06
	Managing demands	Managing demands	0.88	0.067	1.40E-39
	MTAG-leadership	MTAG-leadership	0.91	0.102	7.61E-19
	**Trait1**	**Trait2**	**Genetic Correlation**	**S.E.**	***P***
**all**	Leadership position	Managing demands	0.57	0.042	3.85E-42
**female**	Leadership position	Managing demands	0.56	0.063	3.80E-19
**male**	Leadership position	Managing demands	0.63	0.075	7.04E-17

Supplementary Table 11. Distribution of senior leadership position and genomic control λ for GWAS results in three datasets

Study	Non-leaders	Leaders	All	Genomic control λ
UKB discovery cohort	140,536	2,275	142,811	1.005
UKB follow-up cohort	19,236	1,032	20,268	1.009
Add Health Study	3,185	527	3,712	1.010
All samples	162,957	3,834	166,791	1.026

For all samples, meta-analysis was performed to combine summary statistics from the individual GWAS in each cohort using the MTAG software.

Supplementary Table 12. Constructing PGS for leadership in UKB follow-up cohort

Method	*Pt* cut-off to generate PGS	Pseudo-R2 (cov+ PGS)	Pseudo-R2 (PGS)	*P*-value	# SNP included
PRSice (*9*)	$p_t < 1$	0.0416	4.27E-04	3.27E-04	467926
	$p_t < 0.05$	0.0413	2.02E-04	7.16E-03	54110
	$p_t < 1 \times 10^{-3}$	0.0410	4.85E-05	1.05E-02	1913
	$p_t < 1 \times 10^{-5}$	0.0413	2.33E-04	3.51E-01	30
Lassosum (*10*)	N.A.	0.0413	3.12E-04	0.0107	17037

Sample size in the UKB follow-up cohort, N=22,875 (3639 leaders vs. 19236 non-leaders)
GWAS result from MTA-leadership in UKB discovery sample was used for PGS construction.
The covariates include age, sex, array factors, and top 20 PCs. Pseudo-R^2 was calculated using the McFadden test. *P* - model fitting *p*-values for PGS.
Tuning parameter of Lassosum: lambda = 0.0010 and best.s = 0.2

Supplementary Table 13. Associations between PGS and leadership, or senior leadership position

	Leadership position (N = 22,875) 3,639 leaders /19,236 non-leaders					Senior leadership position (N =20,268) 1,032 senior leaders /19,236 non-leaders				
	OR	95% CI		S.E.	*P*	OR	95% CI		S.E.	*P*
		lower	upper				lower	upper		
PGS										
1st quantile	Ref					Ref				
2nd quantile	1.084	0.965	1.217	0.059	0.173	1.073	0.872	1.320	0.106	0.503
3th quantile	1.138	1.014	1.276	0.059	0.028	1.115	0.909	1.369	0.105	0.297
4th quantile	1.067	0.950	1.198	0.059	0.275	1.148	0.936	1.408	0.104	0.185
5th quantile	1.215	1.084	1.363	0.058	0.001	1.334	1.092	1.629	0.102	0.005
Male	2.876	2.668	3.100	0.038	< 2e-16	3.778	3.288	4.341	0.071	< 2e-16
Age	0.986	0.980	0.991	0.003	0.000	0.990	0.980	1.000	0.005	0.052
Array	1.035	0.920	1.164	0.060	0.568	1.112	0.902	1.371	0.107	0.319

Polygenic score (PGS) was equally grouped into 5 groups based on quantiles. The 1st quantile is the lowest quantile and served as the reference group. PGS was created at *p*-value cut-off of 0.05. Logistica regression models included PGS and other covariates (age, sex, and array factors). Top PCs did not show significant association for outcomes in the model, thus were not included in the final analysis.

Supplementary Table 14. Sources and phenotype description for GWAS results used in the genetic correlation analysis

Phenotypes	PMID	Sample size	Phenotype coding
**Mental health**			
Subjective well-being	27089181	298,420	continuous variable
Overall health rating	27864402	359,681	integer (1,2,3 and 4)
Job satisfaction	UKB data-field 4537	82,190	integer (1 to 6)
Depressive symptom	27089181	180,866	continuous variable and binary variable
Anxiety	26754954	17,310	integer 0 and 2 (those with subsyndromal---ANX=1 were excluded from case-control analysis)
Insomnia	30804565	386,533	binary variable
Alzheimer's	30820047	21,982 cases/41,944 controls	binary variable
Schizophrenia	25056061	36,989 cases/113,075 controls	binary variable
Autism	30804558	18,381 cases/27,969 controls	binary variable
Bipolar disorder	31043756	20,352 cases/31,358 controls	binary variable
ADHD	30478444	19,099 cases/34,194 controls	binary variable
**Physical health & trait**			
Longevity	31413261	11,262 cases/25,483 controls	binary variable (cases were defined as individuals who survive at the age corresponding to the 90th survival percentile, and controls are those whose age at death or at last contact was at or below the age corresponding to the 60th survival percentile)
Number of cancer illness	UKB data-field 134	361,136	integer (0,1,2,3 and4)
Number of non-caner illness	UKB data-field 135	361,141	integer (0,1 and 2)
CAD	28714975	336,924	binary variable
Diabetes	28869590	26,676 cases/132,532 controls	binary variable
BMI	25673413	683,365	continuous variable
Waist-hip ration (WHR)	25673412	224,459	continuous variable
HDL	24097068	196,476	continuous variable
LDL	24097068	196,476	continuous variable
TG	24097068	196,476	continuous variable
**Health Behaviour**			
Smoking initiation	30617275	622,409	binary variable (ever vs. never smokers; cases were defined as individuals who have smoked > 99 cigarettes in their lifetime)

Alcohol intake frequency	30643251	537,349	continuous variable
Physical exercise - SSOE	29899525	350,492	binary variable
**Personal trait**			
Height	25282103	695,648	continuous variable
Agreeableness	21173776	20,669	continuous variable
Conscientiousness	21173776	20,669	continuous variable
Extraversion	21173776	20,669	continuous variable
Openness to experience	21173776	20,669	continuous variable
Neuroticism	29942085	449,484	continuous variable (weighted sum-score of 12 dichotomous items and sum-score of five-point Likert scale with 8 items and 12 items, respectively)
Risk tolerance	30643258	466,571	binary variable
Intelligence	5665562	269,867	continuous variable and binary variable
Educational attainment	30038396	766,345	continuous variable (years of education)
Income	31844048	286,301	integer (5-point scale corresponding to the total household income before tax)

Supplementary Table 15. Genetic correlation between leadership position and personal traits.

Phenotypes	Leadership position						MTAG-leadership					
			95% CI						95% CI			
	r_g	S.E.	Lower	Upper	*P*	FDR	r_g	S.E.	Lower	Upper	*P*	FDR
Height	0.11	0.029	0.054	0.167	1.00E-04	<0.05	0.17	0.026	0.123	0.224	1.19E-11	<0.05
Agreeableness	-0.16	0.330	-0.809	0.484	6.22E-01		0.11	0.270	-0.417	0.640	6.79E-01	
Conscientiousness	0.22	0.182	-0.134	0.579	2.22E-01		0.17	0.153	-0.131	0.470	2.68E-01	
Extraversion	0.51	0.255	0.008	1.009	4.63E-02		0.49	0.214	0.066	0.905	2.32E-02	<0.05
Openness to experience	-0.15	0.145	-0.431	0.139	3.15E-01		0.11	0.120	-0.122	0.349	3.46E-01	
Neuroticism	-0.19	0.040	-0.264	-0.107	3.76E-06	<0.05	-0.25	0.035	-0.315	-0.178	1.37E-12	<0.05
Risk tolerance	0.40	0.047	0.309	0.492	9.15E-18	<0.05	0.32	0.040	0.245	0.400	3.32E-16	<0.05
Intelligence	0.20	0.048	0.106	0.296	3.24E-05	<0.05	0.48	0.039	0.403	0.555	4.62E-35	<0.05
Educational attainment	0.18	0.033	0.114	0.244	6.60E-08	<0.05	0.58	0.029	0.520	0.633	2.29E-88	<0.05
Income	0.53	0.047	0.435	0.619	2.49E-29	<0.05	0.83	0.038	0.752	0.900	2.15E-105	<0.05

Bivariate LDSC regression was applied to calculate genetic correlation r_g. The false discovery rate at <5% was declared as statistical significance.

Supplementary Table 16. Genetic correlation between leadership position and health status.

Phenotypes	MTAG - leadership						MTAG-leadership, partialling out educational attainment						MTAG-leadership, partialling out income					
			95% CI						95% CI						95% CI			
	r_g	S.E.	Lower	Upper	*P*	FDR	r_g	S.E.	Lower	Upper	*P*	FDR	r_g	S.E.	Lower	Upper	*P*	FDR
**Mental health**																		
Subjective well-being	0.26	0.062	0.142	0.385	2.18E-05	<0.05	0.21	0.060	0.088	0.322	5.99E-04	<0.05	0.0005	0.057	-0.111	0.112	9.94E-01	
Overall health rating	0.32	0.039	0.247	0.401	1.60E-16	<0.05	-0.004	0.035	-0.073	0.065	9.09E-01		-0.18	0.036	0.104	0.246	1.24E-06	<0.05
Job satisfaction	0.16	0.079	0.010	0.320	3.71E-02		0.16	0.074	0.015	0.303	3.10E-02		0.01	0.073	-0.149	0.138	9.43E-01	
Depressive symptom	-0.30	0.063	-0.423	-0.178	1.54E-06	<0.05	-0.11	0.064	-0.236	0.014	8.16E-02		0.10	0.058	-0.018	0.211	9.83E-02	
Anxiety	-0.42	0.156	-0.724	-0.113	7.20E-03	<0.05	-0.24	0.133	-0.496	0.024	7.55E-02		-0.08	0.130	-0.333	0.178	5.53E-01	
Insomnia	-0.182	0.046	-0.271	-0.093	6.57E-05	<0.05	0.01	0.045	-0.076	0.101	7.81E-01		0.09	0.045	0.000	0.174	5.02E-02	
Alzheimer’s	-0.15	0.094	-0.334	0.032	1.07E-01		-0.02	0.081	-0.182	0.134	7.67E-01		-0.03	0.078	-0.187	0.119	6.63E-01	
Schizophrenia	-0.08	0.041	-0.159	0.002	5.71E-02		-0.11	0.041	-0.189	-0.029	7.60E-03	<0.05	0.04	0.038	-0.036	0.113	3.11E-01	
Autism	-0.04	0.063	-0.166	0.082	5.06E-01		-0.17	0.062	-0.289	-0.045	7.38E-03	<0.05	0.02	0.057	-0.095	0.130	7.57E-01	
Bipolar disorder	0.14	0.049	0.044	0.235	4.20E-03	<0.05	0.04	0.047	-0.055	0.129	4.28E-01		0.09	0.044	0.008	0.180	3.23E-02	
ADHD	-0.19	0.050	-0.283	-0.087	2.00E-04	<0.05	0.11	0.052	0.009	0.212	3.28E-02		0.19	0.052	0.093	0.295	1.76E-04	<0.05
**Physical health & trait**																		
Longevity	-0.09	0.087	-0.261	0.081	3.04E-01		-0.22	0.078	-0.376	-0.069	4.52E-03	<0.05	-0.23	0.077	-0.377	-0.073	3.63E-03	<0.05
Number of cancer illness	0.03	0.103	-0.168	0.235	7.44E-01		0.09	0.097	-0.103	0.277	3.70E-01		0.10	0.084	-0.065	0.264	2.36E-01	
Number of non-caner illness	-0.16	0.038	-0.232	-0.083	3.57E-05	<0.05	0.02	0.036	-0.052	0.089	5.99E-01		0.12	0.036	0.052	0.192	6.42E-04	<0.05
CAD	-0.04	0.041	-0.124	0.038	3.01E-01		0.14	0.038	0.066	0.214	2.06E-04	<0.05	0.18	0.038	0.103	0.252	2.97E-06	<0.05
Diabetes	-0.07	0.052	-0.175	0.030	1.66E-01		0.06	0.051	-0.038	0.163	2.24E-01		0.07	0.048	-0.020	0.169	1.22E-01	
BMI	-0.01	0.032	-0.073	0.052	7.43E-01		0.16	0.034	0.091	0.223	3.41E-06	<0.05	0.17	0.033	0.107	0.235	1.48E-07	<0.05

Waist-hip ratio (WHR)	-0.13	0.043	-0.211	-0.041	3.60E-03	<0.05	0.05	0.045	-0.040	0.136	2.82E-01		0.10	0.047	0.011	0.193	2.86E-02	
HDL	0.09	0.037	0.018	0.162	1.46E-02	<0.05	0.01	0.051	-0.093	0.107	8.96E-01		-0.03	0.050	-0.124	0.071	5.93E-01	
LDL	-0.11	0.041	-0.193	-0.034	5.00E-03	<0.05	-0.08	0.054	-0.190	0.021	1.16E-01		-0.05	0.052	-0.148	0.056	3.79E-01	
TG	-0.11	0.036	-0.180	-0.041	1.90E-03	<0.05	-0.06	0.056	-0.170	0.051	2.89E-01		0.00	0.049	-0.096	0.096	9.96E-01	
**Health Behaviour**																		
Smoking initiation	-0.06	0.089	-0.233	0.117	5.13E-01		0.12	0.105	-0.090	0.323	2.68E-01		0.09	0.109	-0.120	0.308	3.91E-01	
Alcohol intake frequency	0.11	0.041	0.026	0.186	9.10E-03	<0.05	0.08	0.040	0.004	0.160	3.87E-02		0.05	0.037	-0.026	0.120	2.09E-01	
Physical exercise - SSOE	0.28	0.047	0.188	0.373	3.01E-09	<0.05	-0.01	0.042	-0.096	0.070	7.58E-01		-0.15	0.041	-0.231	-0.070	2.40E-04	<0.05

**Phenotypes**	**Leadership position**						**leadership position, partialling out educational attainment**						**leadership position, partialling out income**					
			95% CI						95% CI						95% CI			
	r_g	S.E.	Lower	Upper	*P*	FDR	r_g	S.E.	Lower	Upper	*P*	FDR	r_g	S.E.	Lower	Upper	*P*	FDR
**Mental health**																		
Subjective well-being	0.25	0.076	0.100	0.399	1.10E-03	<0.05	0.23	0.073	0.086	0.373	1.75E-03	<0.05	0.08	0.070	-0.059	0.217	2.62E-01	
Overall health rating	0.07	0.045	-0.015	0.159	1.05E-01		0.03	0.042	-0.049	0.115	4.26E-01		-0.25	0.044	0.163	0.334	1.30E-08	<0.05
Job satisfaction	0.20	0.091	0.022	0.379	2.75E-02		-0.20	0.089	-0.374	-0.024	2.58E-02		0.10	0.087	-0.269	0.072	2.59E-01	
Depressive symptom	-0.17	0.075	-0.317	-0.022	2.45E-02		-0.11	0.076	-0.259	0.039	1.49E-01		0.09	0.072	-0.055	0.227	2.31E-01	
Anxiety	-0.31	0.167	-0.640	0.016	6.21E-02		-0.27	0.158	-0.574	0.043	9.22E-02		-0.10	0.156	-0.409	0.201	5.04E-01	
Insomnia	-0.041	0.053	-0.144	0.063	4.41E-01		0.02	0.053	-0.083	0.126	6.89E-01		0.13	0.054	0.026	0.237	1.46E-02	<0.05
Alzheimer’s	-0.06	0.108	-0.275	0.149	5.61E-01		-0.02	0.097	-0.207	0.173	8.61E-01		0.01	0.095	-0.173	0.198	8.99E-01	
Schizophrenia	-0.11	0.048	-0.206	-0.018	1.97E-02		-0.12	0.049	-0.213	-0.022	1.58E-02		-0.03	0.046	-0.125	0.056	4.57E-01	
Autism	-0.15	0.073	-0.294	-0.007	4.02E-02		-0.19	0.073	-0.330	-0.045	9.72E-03	<0.05	-0.11	0.069	-0.245	0.025	1.10E-01	
Bipolar disorder	0.09	0.056	-0.016	0.203	9.57E-02		0.06	0.056	-0.048	0.171	2.72E-01		0.06	0.053	-0.040	0.169	2.26E-01	
ADHD	0.04	0.059	-0.077	0.153	5.16E-01		0.13	0.059	0.016	0.247	2.54E-02		0.28	0.058	0.168	0.395	1.23E-06	<0.05

**Physical health & trait**																		
Longevity	-0.22	0.098	-0.415	-0.031	2.27E-02		-0.26	0.093	-0.440	-0.075	5.76E-03	<0.05	-0.30	0.092	-0.484	-0.125	8.96E-04	<0.05
Number of cancer illness	0.12	0.125	-0.121	0.368	3.21E-01		0.14	0.120	-0.094	0.376	2.40E-01		0.17	0.111	-0.051	0.384	1.33E-01	
Number of non-caner illness	0.02	0.045	-0.073	0.103	7.37E-01		0.07	0.044	-0.017	0.156	1.14E-01		0.19	0.043	0.109	0.277	6.13E-06	<0.05
CAD	0.11	0.048	0.013	0.199	2.56E-02		0.17	0.046	0.078	0.259	2.47E-04	<0.05	0.25	0.046	0.160	0.339	4.74E-08	<0.05
Diabetes	0.01	0.062	-0.114	0.131	8.91E-01		0.05	0.062	-0.072	0.172	4.19E-01		0.10	0.060	-0.016	0.221	8.86E-02	
BMI	0.11	0.035	0.042	0.181	1.70E-03	<0.05	0.17	0.039	0.097	0.250	8.45E-06	<0.05	0.23	0.038	0.159	0.309	1.20E-09	<0.05
Waist-hip ratio (WHR)	0.01	0.050	-0.093	0.104	9.12E-01		0.06	0.054	-0.042	0.170	2.37E-01		0.16	0.055	0.050	0.266	4.07E-03	<0.05
HDL	0.02	0.044	-0.069	0.102	7.13E-01		0.00	0.061	-0.117	0.121	9.72E-01		-0.06	0.059	-0.176	0.055	3.06E-01	
LDL	-0.09	0.048	-0.184	0.003	5.69E-02		-0.09	0.065	-0.218	0.036	1.58E-01		-0.04	0.064	-0.169	0.080	4.81E-01	
TG	-0.05	0.043	-0.132	0.038	2.81E-01		-0.06	0.067	-0.192	0.072	3.71E-01		0.02	0.062	-0.105	0.140	7.81E-01	
**Health Behaviour**																		
Smoking initiation	0.02	0.106	-0.187	0.229	8.43E-01		0.09	0.128	-0.164	0.338	4.98E-01		0.13	0.130	-0.124	0.385	3.15E-01	
Alcohol intake frequency consumption	0.11	0.048	0.015	0.203	2.35E-02		0.10	0.049	0.005	0.195	3.92E-02		0.07	0.047	-0.021	0.162	1.33E-01	
Physical exercise - SSOE	0.08	0.054	-0.027	0.186	1.42E-01		-0.01	0.051	-0.111	0.088	8.15E-01		-0.20	0.050	-0.295	-0.099	8.19E-05	<0.05

ADHD – Attention Deficit Hyperactivity Disorder, CAD – Coronary Artery Disease, BMI – Body Mass Index, WHR – Whist-hip Ratio, HDL – High-density Lipoprotein, LDL – Low-density lipoprotein, TG – Triglycerides.

Reference

1. K. M. Harris *et al.*, Cohort Profile: The National Longitudinal Study of Adolescent to Adult Health (Add Health). *International Journal of Epidemiology* **48**, 1415-1415k (2019).
2. P. Herd, D. Carr, C. Roan, Cohort profile: Wisconsin longitudinal study (WLS). *International journal of epidemiology* **43**, 34-41 (2014).
3. C. Bycroft *et al.*, The UK Biobank resource with deep phenotyping and genomic data. *Nature* **562**, 203-209 (2018).
4. H. M. Highland, C. L. Avery, Q. Duan, Y. Li, K. M. Harris, Quality control analysis of Add Health GWAS data. *Carolina Population Center, University of North Carolina at Chapel Hill, Chapel Hill, NC*, (2018).
5. J. Yang, S. H. Lee, M. E. Goddard, P. M. Visscher, GCTA: a tool for genome-wide complex trait analysis. *The American Journal of Human Genetics* **88**, 76-82 (2011).
6. P. Turley *et al.*, Multi-trait analysis of genome-wide association summary statistics using MTAG. *Nature Genetics* **50**, 229-237 (2018).
7. T. M. Glomb, J. D. Kammeyer-Mueller, M. Rotundo, Emotional labor demands and compensating wage differentials. *Journal of Applied Pychology* **89**, 700-714 (2004).
8. S. Lee, N. Wray, M. Goddard, P. Visscher, Estimating missing heritability for disease from genome-wide association studies. *The American Journal of Human Genetics* **88**, 294-305 (2011).
9. J. Euesden, C. M. Lewis, P. F. O'Reilly, PRSice: polygenic risk score software. *Bioinformatics* **31**, 1466-1468 (2015).
10. T. S. H. Mak, R. M. Porsch, S. W. Choi, X. Zhou, P. C. Sham, Polygenic scores via penalized regression on summary statistics. *Genetic Epidemiology* **41**, 469-480 (2017).
